# Enhancing traceability and trust in the sugarcane supply chain using a blockchain-based framework with smart contracts and tokenization

**DOI:** 10.1371/journal.pone.0352021

**Published:** 2026-08-04

**Authors:** Muhammad Shoaib Farooq, Junaid Nasir Qureshi, Haroon Abdul Waheed, Muhammad Asad Khan, Uzma Farooq, Ali Shahaab

**Affiliations:** 1 Department of Artificial Intelligence, University of Management and Technology, UMT, Lahore, Pakistan; 2 Department of Computer Science, Bahria University, BULC, Lahore, Pakistan; 3 Faculty of Information Technology and Computer Science, Department of Software Engineering, University of Central Punjab, UCP, Lahore, Pakistan; 4 Cardiff School of Technologies, Cardiff Metropolitan University, Cardiff, United Kingdom; University of Sannio, ITALY

## Abstract

Sugarcane crop is used for making several eco-friendly food products, making it one of the most important crops in the world. Issues in how sugarcane gets from the field to the factory like hidden dealings and fake shortages, really hurt farmers’ earnings and even national economies. The way things are currently managed varies a lot in openness, making it tough for authorities to catch illegal exports or guarantee farmers get paid fairly. To tackle these problems, this article suggests a new system built on blockchain technology. It is designed to make the sugarcane supply chain more traceable and trustworthy. We’re introducing a special digital currency called Sugar Coin (SC) to follow transactions between everyone involved. Along with this, we have a full system for handling these transactions using smart contracts. We’re also using something called Inter Planetary File Systems (IPFS) to securely store private information from farmers and retailers. This helps keep data safe and easily accessible. Tests show that our suggested framework works better than other supply chain projects when it comes to how quickly blocks are processed, how many transactions it can handle, and the costs involved. This research makes the sugarcane world more reliable and open, and it also suggests a lasting economic plan by launching SugarCoin through something called an Initial Coin Offering (ICO).

## Introduction

**Fig 1:**
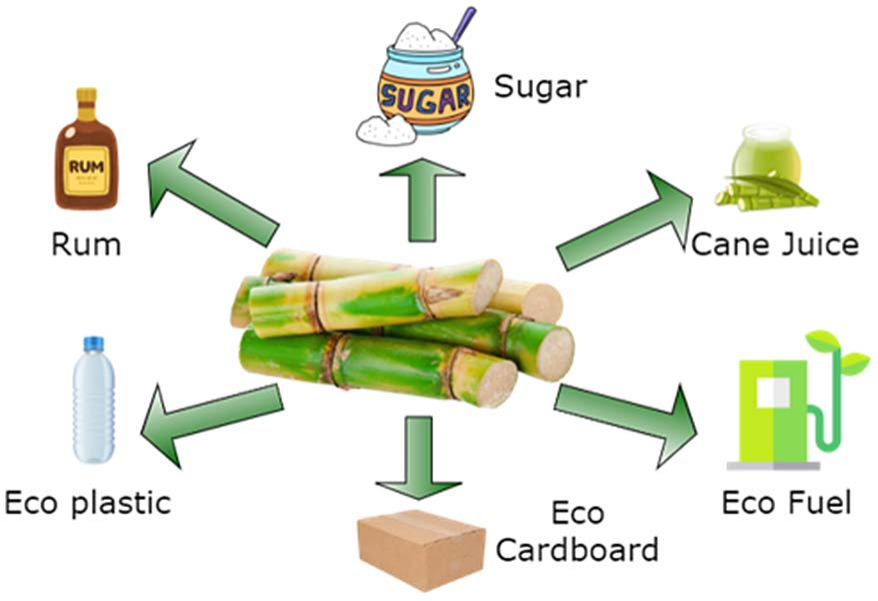
Sugarcane products.

Monitoring Agricultural food products from the production to the consumption phase is a critical aspect of food safety and a smooth supply chain [1,2]. The risks of contamination and food safety concerns have increased, leading to growing demand for enhanced traceability in the supply chain [3]. Furthermore, agricultural products are being traded across borders, which has increased the demand for proper traceability of products to comply with each country’s regulations. Agricultural product traceability includes various types of information that are collected by multiple data sources and information exchanges in the supply chain [4,5]. The sugarcane is a species of tall, perennial grass that is cultivated for its stalks, which are rich in sucrose. Sucrose accounts for 60 percent of worldwide sugar production, while the remaining 40 percent is produced from sugar beets [4]. Some countries use sugarcane for bioethanol production [6,7]. Sugarcane is grown in more than 90 countries, with Brazil, India, China, Thailand, and Pakistan being the top five producers [8]. Sugarcane has also been used to produce other eco-friendly by-products, such as cane juice, cardboard, paper, rum, fuel, and plastic, as shown in Fig 1.

**Fig 2:**
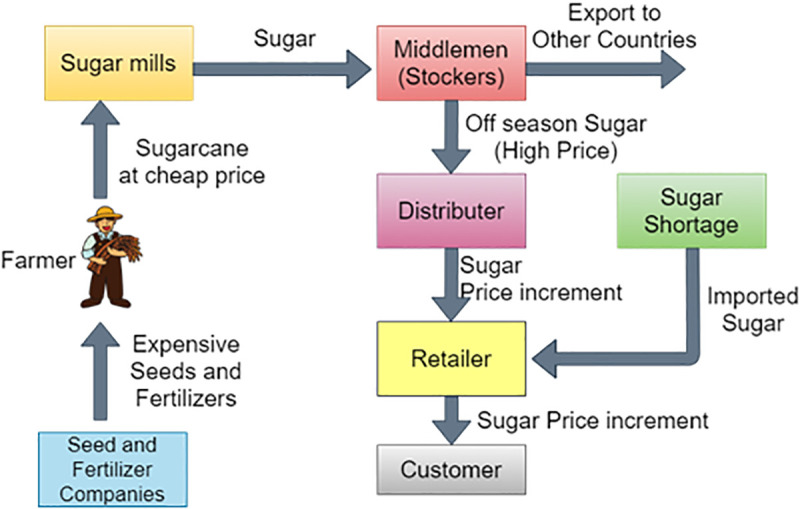
Current supply chain system of sugarcane crop.

The dynamic nature of sugarcane production, processing, and consumption makes tracking and gathering accurate information difficult. This leads to an unexpected increase in the price of the final product of sugarcane and sugar, which has been delivered to the consumer after passing through middlemen between the farmer(producer) and the consumer [6,9]. The seed and fertilizer companies often ask for high prices for basic cultivation needs because of the lack of regulations in the industry [10,11]. Even when regulations do exist, farmers may not be aware of them and end up purchasing expensive seeds and fertilizers [12]. Farmers put in a great deal of effort to grow the crop, but when the sugarcane is ready to harvest, they rush to sell it to sugar mills. At this point, sugar mills have a high supply and low demand, leading to the purchase of sugarcane crops at very low prices, which discourages and demotivates farmers from further cultivating sugarcane [9,13]. During this stage, some businesses may also get involved in buying cheap sugarcane products from sugar mills and exporting them at higher rates [14,15]. Some unscrupulous traders may store sugarcane products during the crop season and then sell them at high prices during the off-season. The government may import sugar from other countries at high rates due to a shortage of sugarcane products during the off-season [16,17]. Although some countries have well-defined rules and regulations, the poor traceability of the supply chain process often makes it nearly impossible to control prices with the current system, as illustrated in Fig 2.

The main reason for the aforementioned problems is a deficiency in transparency in the sugarcane management process. Farmers are discouraged by the very low amount of money they earn in return for their hard work. Customers have to pay high prices for sugar, which is a basic human need. The government has to pay for imported sugar and provide subsidies, leading to losses for the overall economy of the country. The current sugarcane crop management process is ineffective in providing trust, transparency, and profits to participants [18]. This highlights the need for a solution to these problems in order to make sugarcane crop management more transparent and reduce sugar prices. One possible solution is a blockchain-based system for transparent management and traceability of sugarcane crops, by removing middlemen and using decentralized contracts [19]. Blockchain technology has the technical ability to decentralize crop data, making it tamper-proof, and capable of covering all types of transactional and digital data, such as crop data, farmer and sugar mill data, seed companies, and retailer data, requiring authenticity. These features make blockchain a perfect solution for sugarcane crop traceability problems. Blockchain is not only secure, but it also has additional layers and applications developed to provide double-checking on all transactional data, which increases participants’ trust in the system and helps to make sugarcane and seed trades more secure. Smart contracts are a key supporting component of blockchain that helps implement business logic and consensus protocols [20].

**Fig 3:**
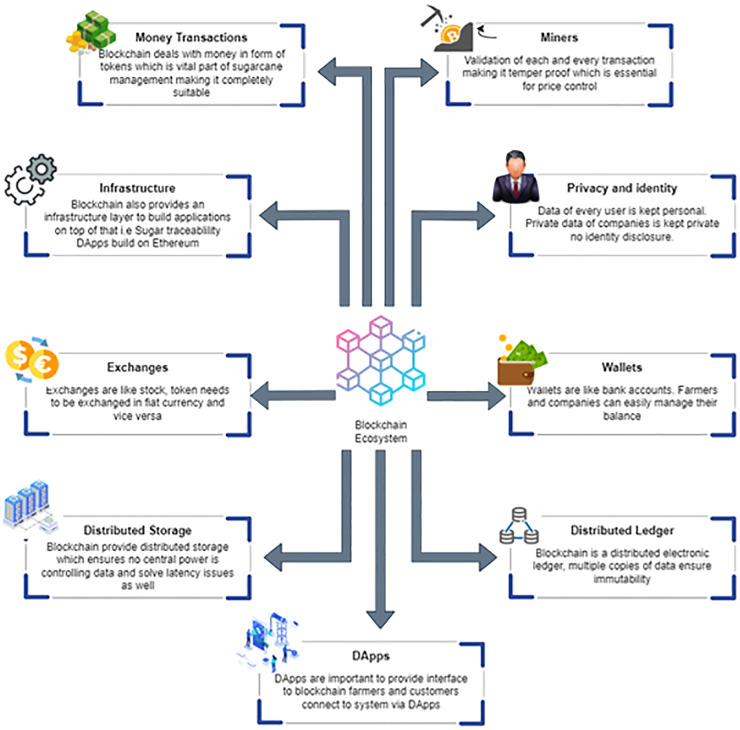
Blockchain ecosystem.

Consensus protocols are the basic rules that provide the backbone for the blockchain by verifying transactions and adding new blocks [21]. Consensus protocols and smart contracts help increase the trust, security, speed, and cost-effectiveness of the blockchain by implementing sugarcane business logic [22]. Due to the distributed nature of the blockchain, it does not allow outside elements to dishonestly access, remove, or modify data, which keeps our sugarcane supply chain system transparent and trustworthy. Once any transaction data is added to the blockchain, it cannot be accessed or changed by any untrusted entity [23]. Data is added to the blockchain by mining blocks, which requires a lot of computational power to solve mathematical puzzles. This process is known as mining, and the nodes that mine new blocks are called miners [24]. Multiple miners in different locations participate in mining blocks, and the distributed nature of miners makes the blockchain un-breechable. However, if a single entity owns 51 percent or more mining power of a blockchain, then it can manipulate it. This attack factor is known as a 51 percent attack. To mitigate this attack, miners should be distributed across multiple locations and not belong to a single entity [25]. Blockchain technology ensures that transactions are confirmed quickly with feedback, making sugarcane traceability more easy and transparent. Anyone can access data and trace it back to its origin. Therefore, the blockchain is an appropriate technology for acquiring a sugarcane traceability system due to its decentralized nature and security features. The blockchain ecosystem with its features is presented in Fig 3. Current supply chain systems are facing security and transparency issues, middle exploitation, and fluctuation in prices. To overcome these challenges a blockchain-based traceability framework is required that can enhance food safety in any country [26,27]. The sugarcane traceability system relies on multiple components, including participant identities, privacy, wallets and exchanges, transactions, decentralized applications, distributed storage, ledger, and miners. The blockchain ecosystem contains wallets and exchanges that enable compatibility of cryptocurrency flow and management, embedding transaction data with timestamps in an immutable distributed ledger. Furthermore, the blockchain provides additional features such as infrastructure to build DApps that can communicate with farmers, other participants, and users more easily. Other constituents of the blockchain, including distributed ledger and storage, make it a more compatible solution for sugarcane traceability, providing greater control and transparency of data for participants [28,29].

This study advances the state of research on blockchain-enabled agricultural supply chains through several distinct and original contributions that go beyond existing literature: 1. First integrated framework for sugarcane traceability and price control To the best of our knowledge, this is among the first studies to jointly address end-to-end sugarcane supply-chain traceability and transaction price control within a single decentralized blockchain framework. Prior works primarily focus on traceability alone or provide conceptual discussions without integrating economic and governance mechanisms. 2. Novel coupling of traceability with a token-based settlement model Unlike existing agricultural blockchain solutions that record provenance without addressing economic flows, this paper introduces Sugar Coin (SC) as a native, stable-value settlement token explicitly designed for agricultural commodity transactions. The integration of traceability events with escrow-based token settlement represents a new design pattern for regulated agri-commodity markets. 3. Regulatory-aware decentralization rather than purely permissionless design While most prior blockchain supply-chain studies either assume full decentralization or ignore governance entirely, this work proposes a regulatory-aware decentralized architecture. Government supervision is embedded through role-based access control, auditable smart contract events, and authorized access to encrypted records—offering a practical balance between decentralization and regulatory compliance that is largely absent in existing research. 4. Explicit price stabilization and policy-driven token design The proposed SC pricing model goes beyond static token assumptions by introducing policy-controlled mint–burn mechanisms and reserve-backed stabilization concepts. This positions SC closer to a regulated settlement instrument rather than a speculative cryptocurrency, which is a novel contribution in the context of agricultural blockchain systems. 5. Implementation-level specification with experimental validation Many prior studies remain conceptual or architectural. In contrast, this work provides implementation-oriented details, including smart contract pseudocode, execution workflows, encryption strategies, and experimental performance evaluation. This combination of design specification and empirical validation significantly strengthens reproducibility and distinguishes the study from earlier proposals.

Practical implications

- The proposed framework demonstrates how blockchain systems can be operationalized for real agricultural markets, not merely as proof-of-concept traceability tools.
- By reducing reliance on intermediaries and enabling verifiable settlement, the system has the potential to improve farmer compensation, market transparency, and regulatory enforcement in sugarcane supply chains.

Theoretical implications

- The study extends blockchain supply-chain literature by introducing a multi-layered model that integrates provenance, economics, and governance, rather than treating these aspects in isolation.
- It contributes a reference architecture for designing blockchain systems that must operate under regulatory oversight, addressing a gap between decentralized technologies and institutional requirements. Policy and research implications
- For policymakers, the framework illustrates how digital tokens and smart contracts can be aligned with commodity regulation and food security objectives.
- Future research can extend this work toward cross-border trade, adaptive pricing mechanisms, and large-scale field deployments, using the proposed architecture as a foundational model.
- A decentralized, blockchain-based framework for reliable sugarcane traceability.
- The introduction of Sugar Coin (SC), an Ethereum-based token to standardize payments and control price volatility.
- A comprehensive system architecture utilizing IPFS for secure data storage and smart contracts for automated transaction governance.
- Experimental validation comparing the proposed framework against existing solutions in terms of latency and transaction costs.

The main contributions of this article are as follows:

- This study proposes a decentralized blockchain-based reliable, transparent, and secure framework to provide an efficient way of sugarcane crop traceability. It would solve traceability issues and malpractices in the current system.
- A framework has been proposed to get control over the limitations of the existing system of sugarcane farm-to-fork by utilizing blockchain technologies.
- The Sugar Coin as a token has been proposed in this study to control sugar prices. This token is based on the Ethereum smart contract and has discussed token creation, total supply, initial coin offerings, rules for SC buy and sell, and authentication for usage. This would help in controlling prices and wages paid to farmers, employees, and all other actors of the supply chain.
- This article presents details of sugarcane traceability solutions using blockchain technology and system architecture.

The sugarcane industry has been facing various challenges, including fraud, exploitation, and environmental degradation, making it difficult to maintain sustainable and transparent practices in the supply chain. Conventional supply chain management methods have not been able to address these issues effectively, and existing frameworks have often been either too complex or too generic for the specific requirements of the sugarcane industry. The framework has been proposed after conducting extensive research into the existing literature on blockchain-based supply chain management and analyzing the specific needs of the sugarcane industry. We have developed a framework that utilizes the unique features of blockchain technology to provide a more transparent and traceable supply chain. Through the use of the Sugar Coin token, the framework incentivizes sustainable practices and creates a reliable record of all transactions on the blockchain. This approach promotes greater accountability and transparency throughout the supply chain, which is essential for ensuring the long-term sustainability of the sugarcane industry. In section 2 related work of other authors has been discussed. Sections 3 and 4 describe in detail the materials and methods used for the proposed system. Section 5 depicts experiments performed and obtained results. Section 6 reflects the conclusion of all work done in this research article.

## Related work

In this section review and highlights of related work found in the literature of blockchain traceability and price control of different food and agriculture supply chains are discussed. Saleh K. et. al. [3] proposed a solution for soya bean traceability by eliminating centralized authority. IPFS file system was used for storing transactions ledger. However, no experiments were made to prove system integrity and framework performance.

Musamih et. al. [10] presented an Ethereum blockchain-based approach for the healthcare supply chain. The framework for the drug supply chain was presented with the help of smart contracts. However, problems like immutability, data privacy, scalability, interoperability, and efficiency arose due to the limitations of blockchain.

Villalva-Cataño et. al. [11] analyzed the causes of high logistic costs by an analysis methodology for the coffee business in Peru. ABC costing analysis methodology was used. This study finds causes and provides solutions. However, problem causes and provided solutions revolve around centralized methods and entities which can lead to trust issues. Any decentralized approach can be made for better and trustless results.

Pandey et. al. [13] analyzed the Additive Manufacturing supply chain and proposed three models to mitigate possible attack vectors and a new class of cyber-physical risks. All proposed model has testing and good safety approaches. However, all three proposed systems are not decentralized and do not use cryptographic techniques to secure data and its integrity. Hence making it unreliable.

Ferdousi et. al. [14] proposed a distributed supply chain framework to manage US Beef Cattle supply chain. Users, communication, and data are handled when ownership is generated or transferred.

Ekawati, R., et al [26] proposed a system that is based on blockchain technology. The proposed system manages and stores the volume of sugar. The smart contract has been used to observe the transactions that have been performed in the system. The hash code transactions have been deposited to the user’s wallet. All the transaction records have been stored on the blockchain system. However, the blockchain is not transparent for all stakeholders and only visible and transparent to the factory management. Moreover, no proof of system claims has been shown in the experiment.

Ekawati, R., et al [27] discussed the dynamics of the sugar agro-business which is defined by long-term cooperation between actors ranging from farmers to processors. Numerous parties have been involved and have had an impact on the supply chain system consequently disrupting the movement of products, exchange of information, and financing. As a result, the authors proposed the creation of an integrated white crystal sugar agro-industrial supply chain system based on blockchain technology. The objective is to improve competitiveness in ensuring food security and adaptability by proposing a solution to the issue of supply chain miscues that take place from raw materials to finished goods. However, the authors do not perform experiments to prove their claims system’s security and integrity.

Trollman H. et. al [29] investigates how blockchain technology can support the ecological embeddedness of the coffee supply chain. This paper includes the use of a qualitative case study methodology and the identification of gaps in implementation and opportunities for improvement. However, the paper includes a limited focus on only one coffee business in the USA and a lack of clear guidance on how to implement the suggested improvements.

Venkatesh et al. [30] designed Blockchain-based architecture by considering social sustainability in the supply chain area. Social sustainability means creating an ideal work environment, a transparent payment system, a free communication platform, and maintaining employee’s health. Supply chain has become very complicated over the globe due to different companies and individuals therefore social sustainability and social sustainability can be made possible by using Blockchain technology. However, the practical implications of the proposed architecture are missing to develop social sustainability in the supply chain.

Ronaghi et al. [31] introduced a Blockchain-based model for the agricultural supply chain and discussed how Blockchain technology is integrated with other technologies such as IoT and smart contracts to record transactions. However, there is a lack of traceability, trust, vulnerability, and security issues while handling large amounts of agricultural data.

This discussion reveals that a lot of efforts have been carried out in the past for managing agriculture supply chains yet there is no comprehensive decentralized solution available for supply chain traceability. Also, there is a need for a reliable solution for governments to control and track malpractices in the sugarcane supply chain. In this article, we have proposed a blockchain-based framework for sugarcane supply chain transparency and reliability. The novelty of our work is that we provided a secure supply chain solution for sugarcane crops and a solution for authorities to keep track of supply chain transactions. We have proposed a new cryptocurrency token for sugar trades and the traceability of transactions. We have also proposed a decentralized economic model for sugar coin stability and exchange. Table 1 reflects the summary of related work. Table 1 depicts the comparison of related work.

**Table 1:** Related work comparison.

Study	Domain	Blockchain Use	Traceability	Decentralized	Economic / Price Control	Experimental Validation	Key Limitations
Saleh et al. [3]	Soybean	Blockchain + IPFS	Yes	Partial	No	No	No performance or integrity evaluation
Musamih et al. [10]	Healthcare	Ethereum + smart contracts	Yes	Partial	No	Limited	Scalability, privacy, interoperability issues
Villalva-Cataño et al. [11]	Coffee logistics	No blockchain	Partial	No	No	Yes	Centralized, trust-dependent
Pandey et al. [13]	Additive manufacturing	Security models	No	No	No	Yes	No cryptographic or decentralized design
Ferdousi et al. [14]	Beef cattle	Distributed framework	Partial	Partial	No	Limited	No full traceability validation
Ekawati et al. [26]	Sugar	Blockchain + smart contracts	Partial	Partial	No	No	Limited transparency, no experiments
Ekawati et al. [27]	Sugar agro-industry	Blockchain-based	Partial	Partial	No	No	No security or integrity validation
Trollman et al. [29]	Coffee sustainability	Conceptual blockchain use	Partial	Partial	No	Qualitative	Single case, no implementation
Venkatesh et al. [30]	Supply-chain sustainability	Blockchain architecture	Conceptual	Partial	No	No	Lacks practical implementation
Ronaghi et al. [31]	Agriculture	Blockchain + IoT	Partial	Partial	No	Limited	Security and scalability concerns
**This study**	Sugarcane	Blockchain + IPFS + smart contracts	**Yes**	**Yes**	**Yes (Sugar Coin)**	**Yes**	—

Overall, existing research highlights the potential of blockchain in agricultural supply chains but reveals significant gaps. Most prior works lack comprehensive decentralization, experimental validation, economic mechanisms for price control, or explicit support for regulatory oversight. In particular, no prior study provides an integrated, experimentally evaluated framework for sugarcane supply-chain traceability combined with transaction settlement and price control, motivating the approach proposed in this study.

## Materials and methods

The theoretical framework for this research has been developed based on the principles of distributed systems, supply chain management systems, and Blockchain technology by following the key concepts including transparency and trust in Blockchain. Additionally, to enhance efficiency and reliability among supply chain systems deployed smart contract and cryptographic techniques for reliable and secure data storage [32]. Furthermore, the economic models, architectures, and sugar coins are considered while designing the proposed framework.

### Proposed framework

In this article, a method has been proposed to make a secure, efficient, and transparent sugarcane traceability system to track sugar from farm to fork using state of art technology known as the blockchain. The framework is developed based on a thorough analysis of the issues in the sugarcane supply chain and the potential for blockchain technology to address traceability and corruption issues. The development process involved extensive research on existing literature in the area of blockchain-based supply chain management and an analysis of the specific needs of the sugarcane industry. The framework has been designed to tailor the unique requirements of this sector, which includes the use of the Sugar Coin token for sustainable practices and providing a transparent and traceable record of all transactions on the blockchain. This technology makes a transparent system for retailers to get quality sugar at low prices with complete traceability of sugar production processes. Once a farmer purchases seeds from a seed company for sugarcane production, all data and payment details are stored in the blockchain, and business rules as smart contracts are invoked, which are secured by the proof-of-work consensus protocol of the blockchain. Any payment or data stored in the blockchain through smart contracts cannot be tampered with, altered, or deleted. Before buying sugar from any distributor, a retailer can track its seed origin, production farms, crop yield dates, distribution network details, sugar mill production details, sugar distribution network details, retailer purchase information, price details, and all processes followed using the blockchain distributed ledger. Payment made for the purchase of sugarcane is considered pending until it is approved by authorized sources to control the prices of sugar. The government can regulate transactions with public blockchain data. If any unusual event occurs, the authorities can take action against malicious actors.

**Fig 4:**
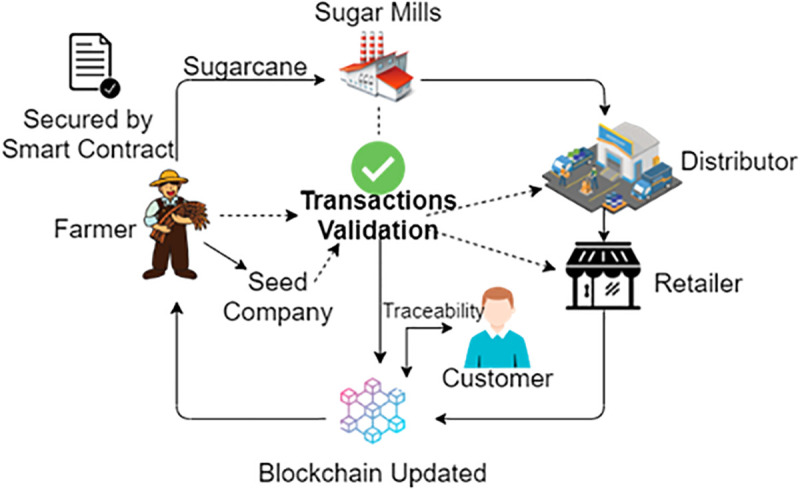
Proposed framework for sugarcane crop.

Fig 4 illustrates the contribution of blockchain technology in improving the current sugar production system. The proposed framework shown in Fig 4.

#### Proposed framework architecture.

**Fig 5:**
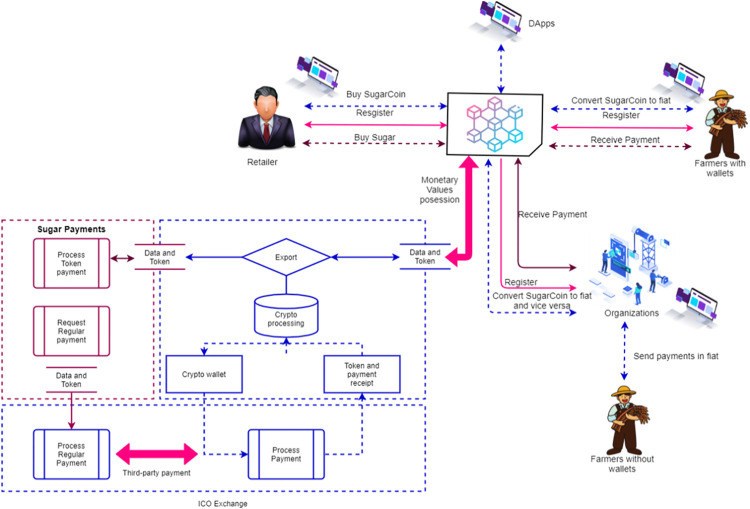
Workflow of proposed sugarcane crop framework.

Stakeholders of the framework are users including farmers, seed companies, sugar mills, distributors, retailers, retailers, and Government organizations. The main components of the framework are decentralized application (DApp) which depends on blockchain network, web platform for user interaction, Digital Storage, IPFS, and smart contract. Every user registers by uploading data on the Sugar management system via DApp or the web and gets a unique public address. Each public address assigned is different for each user and helps identify each entity over the network. IPFS works as an intermediate medium for data transformation between DApp and digital storage. The proposed solution has three main actors farmers, retailers, and organizations which may connect with the Sugar Management System (SMS) through Web or mobile application using a blockchain network. There are multiple admins included as well and are considered as users of SMS which tend to manage and authorize processes according to their authorities and levels. The Sugar Management platform is a DApp that includes initial coin offerings (ICO), ICO exchange, payment gateway integrations including local and digital currencies, and multiple digital wallets to manage different coins and tokens [32,33]. The Sugar Management Platform utilizes IPFS to share and store users’ information and statistical values using smart contracts. IPFS transfers information between Digital storage and distributed peer networks which are controlled by users via their identity keys, and public addresses. Fig 5 illustrates the architecture of the proposed framework, the workflow of the framework.

A retailer has an account on Dapp that has a digital wallet associated with it from where he can purchase sugar and money is then transferred to the farmer with each entity between them getting their fixed commission like sugar mills and retailers. Once retailers buy sugar the blockchain ledger adds an entry including a traceability record from farm to fork. Authorities get notifications for the price at which sugar is sold to retailers. The important thing to note is that not all farmers have digital wallets so in such cases organizations with digital wallets participate they help farmers convert digital money into cash.

#### Economic model.

To propose an economic model for transparent, secure, and auditable sugar management and price control for sugar by removing middlemen, a new cryptocurrency has been proposed called SugarCoin. The total supply of SuguarCoin is 500 million, each has a fixed price of 100 USD. There is no fixed 100 USD purchase limit, any user can buy 10 USD or other worth of token as desired as the token can be fractionalized like 0.1 SC for 10 USD. SugarCoin could be bought using local currencies like PKR, USD, EUR, etc. or cryptocurrencies like BTC, ETH, BNB, etc. SugarCoin, being a part of a decentralized blockchain platform, could be bought without paying exchange fees and tax providing benefits to retailers who can get an equal amount of tokens that they had paid for. Once retailers bought SugarCoin then equivalent balance was added to their wallets. Retailers could use these tokens to buy sugar from distributors without paying any tax and fee to exchanges and third-party applications with a 100percent conversion rate between SugarCoin and other currencies. SugarCoin was deployed as an ERC 20 token on Ethereum blockchain and its initial distribution was done using ICO. SugarCoin token details including maximum supply, token type, price, and symbols. Table 2 shows the Focus Area and Technology Used. If a fee of 1 percent is charged by SC smart contract then fee can be calculated through formula where totalAmount is price for which we want to calculate fee. Fee = (Total Amount*1) / 100.

**Table 2:** Focus area and technology used.

Author / Study	Focus Area	Technology Used	Limitations
Saleh et al. [3]	Soybean Traceability	Blockchain, IPFS	No performance experiments conducted.
Musamih et al. [10]	Healthcare / Drugs	Ethereum Smart Contracts	Issues with scalability and data privacy.
Ekawati et al. [26]	Sugar Volume	Blockchain	Lack of transparency for all stakeholders; limited to factory management.
Trollman et al. [29]	Coffee Supply Chain	Blockchain Case Study	Limited focus on a single business; lacks implementation guidance.
Proposed Framework	Sugarcane Traceability	Binance Smart Chain, IPFS, Sugar Coin	Addresses scalability, price control, and privacy via hybrid storage.

There are two ways for the farmer to receive payments in return for their sugarcane crop yield sold. One way is for farmers who have digital wallets such farmers can receive direct payments in SugarCoin and then use an ICO exchange to convert these tokens to fiat currency. Another way to receive payments from farmers is to contact organizations assigned by the government. These organizations have digital wallets and can receive SugarCoin payments and convert it to fiat using ICO exchange and then deliver fiat payments to Farmers. In each case when farmers receive payments then proof of payment as an image or scanned receipt is delivered back to the blockchain for surety for the accurate price paid to the farmer.

**Fig 6:**
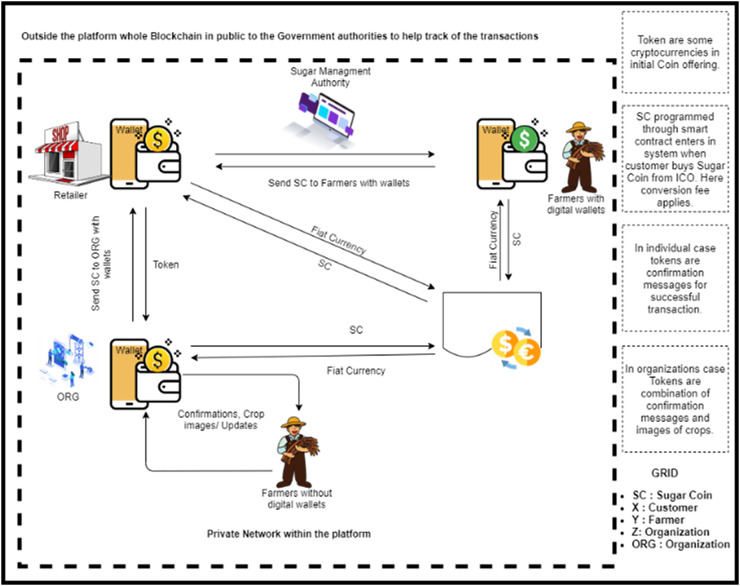
Economic model of proposed framework.

The blockchain is public in nature for multiple stakeholders. Blockchain data that is stored on IPFS is private and accessible only for stakeholders such as farmers, retailers, sugar mills, and distributors and also publically available for the government to verify, authenticate, and audit. Data that is stored on IPFS in encrypted form and can only be accessed using the private keys of respective stakeholders. Fig 6 demonstrates the proposed economic model.

#### Token distribution.

Initial minted SugarCoin were distributed as follows: seventy percent (70) percent of initial mint were allocated for ICO from where retailers, sugar mills, distributors, farmers, and other stakeholders bought coins. While, 20 percent of initially minted coins were allocated for management, marketing, market making, trading, liquidity, and key accusations. The remaining 10 percent of tokens were assigned for more advancements in the ecosystem and system reserves for managing uncertain issues. SugarCoin’s first mint was the generation of all SugarCoin possible there will be no new coins ever generated.

#### Conversion fee.

To make SugarCoin sustainable for a long time some fees were defined. For the management of SugarCoin and other expenses, an exchange fee was defined. This fee varies from one exchange like Binance etc. to others. This fixed fee is charged as defined in the rules. It was charged when SugarCoin was sold or bought. Each transaction including SugarCoin for sending from one party to another would include a small mining fee that would be paid to management authorities to keep the system running in the long term.

#### Workflow of proposed sugarcane crop framework.

**Fig 7:**
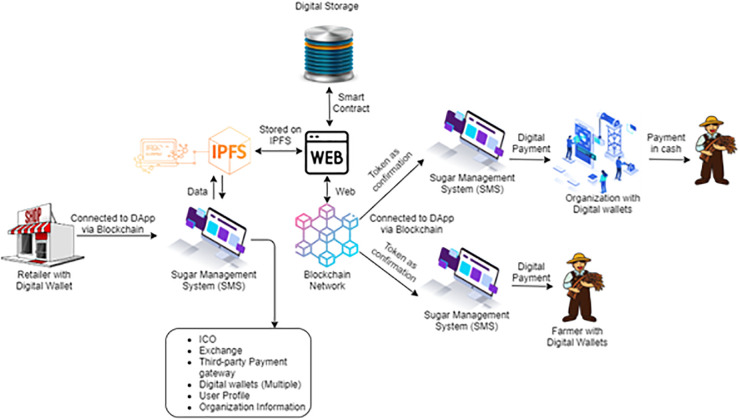
Proposed system architecture for sugarcane crop.

Fig 7 elaborates the architecture workflow of the proposed sugarcane crop framework. Users connect with DApp directly, a sugar management system. Every user of the system has their profiles on DApp where they have a public address and wallet with options to manage their profile. This platform has an exchange and ICO running at the backend. For buying SC retailers made payments through many bank incorporations and credit card (CC) payments, and exchanges that were connected to ICO converted payments to SC. The retailer received UTXOs after a complete cycle in his wallet which could be sent directly to distributors and sugar mills. Sugar mills and distributors could also use the exchange to convert SC to fiat currency and vice versa. Organizations also use exchanges for the conversion of SC to fiat and pay farmers with no wallets. Sugar Coin is exchanged with other currencies by utilizing a Decentralized Exchange of DEX. This DEX provides unlimited liquidity and resources to convert between multiple tokens. Admin user dealt with smart contract management, user authentication, and token distribution policies. Smart contract controls exchange and ICO connections. SC price rates on exchanges against multiple currencies were defined according to smart contracts.

#### Layered structure of the proposed framework.

**Fig 8:**
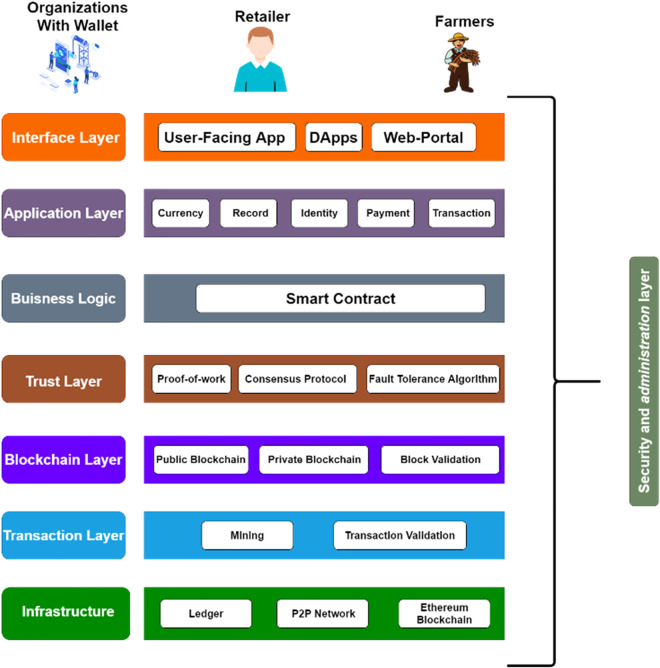
Layer architecture of proposed framework.

The architecture of our proposed framework is layered-based. It includes eight different layers including the application layer, infrastructure layer, interface layer, business logic layer, security and administration layer, blockchain layer, transaction layer, and trust layer. Each layer’s details is as follows:

1. Interface layer: This layer includes user interaction applications, web applications, and dApps. This layer provides an interface for farmers, retailers, sugar mills, customers, and other stakeholders to interact with the sugar management system. This layer provides an interface for the initialization of all processes.
2. Application layer: The application layer encapsulates records, payment data, identity verification, and transaction metadata. This layer is responsible for secure communication between interface layers with a business logic layer in the form of a smart contract.
3. Business logic layer: This layer contains smart contracts that deal with all regulations, rules, terms, user interaction criteria, and all role scenarios. This layer includes all rules of invocation
4. Communication, and execution, hence it is considered as an active database of smart contracts.
5. Trust Layer: This layer encompasses security analysis through smart contract audits, formal verifications, and consensus protocols such as byzantine fault tolerance algorithms, proof-of-stake, and proof-of-work. The trust layer is responsible for implementing consensus algorithms on all new blocks and transactions and storing execution results on the blockchain layer.
6. Blockchain layer: This layer stores information on block status and distrusted nodes. The blockchain layer is also responsible for storing the distributed ledger add all transaction records of farmers, sugar mills, retailers, and all other stakeholders against their public addresses.
7. Transaction layer: This layer deals with all sugar coin transactions happening between farmers, retailers, sugar mills, seed companies, and all other stakeholders of the sugar management system.
8. Infrastructure layer: This layer consists of Peer to Peer (P2P) network to forward, verify, and distribute transaction data mapped on Ethereum Blockchain. This layer is also responsible for distributed network mechanisms, communication, and verification. Once a transaction gets approved from one node then it is distributed to all other nodes which check it according to predefined rules and if it gets verified it is stored on blockchain.
9. Security and administration layer: This layer performs an important role as it provides security against multiple attacks including but not limited to 51 percent of attacks. This layer works in parallel to the system and has multiple protocols and security checking mechanisms to maintain sugar management system integrity against malicious actors. Users such as farmers, sugar mills, retailers, and seed companies interact through applications and web portals in the interface layer to perform transactions. Application layers connect user’s transactions with smart contracts which imply business rules and logic and forward transactions to trust layers where transactions are stored in blockchain through proof-of-work and other consensus protocols. The blockchain layer stores these transactions in a public ledger against user’s private and public keys after block validations through transaction layers. Infrastructure and security layers help maintain system integrity by implementing security checks and multiple protocols. The layered structure of the proposed framework is presented in Fig 8.

#### Smart contract.

Smart contracts are finite pieces of code stored on the blockchain as transaction data. The user can interact with this code by calling its functions for new transactions. The proposed framework was built on sugarcane product traceability using smart contracts. The smart contract was accessed for testing through a simple Decentralized Application (DApp) [34,35]. This DApp was a functional website that was built through HTML, CSS, and JAVASCRIPT languages.

Sugarcane products traceability Smart contract: Sugarcane products traceability smart contract starts with a declaration of two Struct type variables. The first one is the struct of SugarcaneProduct which contains id, name, quantity, other features, numbers of traces, number of properties, traces of sugarcane product, an array for properties of sugarcane products, address of product adder (farmer), global id and encrypted hash of document uploaded on IPFS. The second struct consisted of ProductTrace which contains properties like id, id_sugarcaneProduct, location, current temporary owner, timestamp, and trade details to up-loader details. The next step for adding a sugarcane product is Sugar. id, name, quantity, number of traces, number of properties, and product global id that are considered as properties for the product. This function is called by farmers when they add a new sugarcane product. This function checks whether data is uploaded by the correct authority person. The smart contract also processes a function for adding traces to sugarcane products. This function is used for statistical analysis and traceability functions. This feature allows users to track location traces, and previous and current owners with the timestamp of each transfer.

#### Transactions.

**Fig 9:**
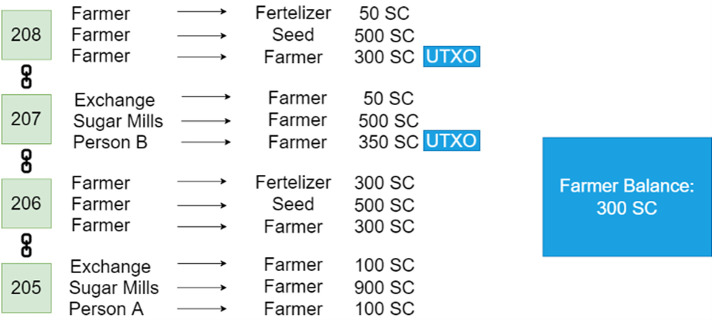
Sugarcoin UTXO of Farmer wallet.

In the proposed framework transactions work on unspent transaction output (UTXO). Each wallet has its own unspent sugar coins which stay in the farmer’s wallet after a transaction. Fig 9 illustrates the Sugarcoin UTXO of the farmer wallet. It also shows that if a farmer receives 100 SC from an exchange, 900 SC from a sugar mill, and a person A sends him 100 SC then the total UTXO in the farmer’s wallet will be 1100. The SC in block 205 is not utilized by farmers. In the next 206 blocks, the farmer spends 300 SC to buy fertilizers and 500 SC for buying seeds from seed companies. Now in block 206, UTXO is 300 SC which is sent back to the farmer’s wallet and the total wallet balance at block 206 is 300 SC. In next block 207, the farmer again receives 50 SC from Exchange, 500 SC from the sugar-mill, and 350 SC from person B. In block 207 UTXO of the farmer’s wallet is 900 SC.

In the next block 208, the farmer spends 50 SC for purchasing fertilizers, 500 SC for seeds, and the remaining 300 SC are sent back to his wallet. At block 208 balance of the farmer’s wallet is 300 SC.

#### Attacks and security.

The proposed framework deals with the most common attack of faulty nodes that validate transactions. In this type of attack, some malicious actors try to implement bad data or illegal funds transfers. The proposed framework deals with such problems through the Byzantine Fault Tolerance consensus algorithm. The Byzantine Fault Tolerance provides the ability to make all nodes come to one conclusive decision even if some of the nodes become faulty and malicious [36,37]. The Byzantine Fault Tolerance mechanism helps in maintaining original blockchain data.

#### Smart contract algorithm.

Contract A: ParticipantRegistry (registration + roles)

contract ParticipantRegistry [

enum Role [FARMER, MILL, TRANSPORTER, DISTRIBUTOR,

RETAILER, REGULATOR]

struct Participant [

Role role;

bool active;

]

mapping(address => Participant) public participants;

address public admin;

constructor() [

admin = msg.sender;

]

function register(address user, Role role) external [

require(msg.sender == admin, "Only admin");

participants[user] = Participant(role, true);

]

function deactivate (address user) external [

require(msg.sender == admin, "Only admin");

participants[user].active = false;

]

function requireRole(address user, Role role) external view [

require(participants[user].active, “Inactive”);

require(participants[user].role == role, "Wrong role");

]

]

Contract B: SugarCoin (token issuance + transfers)

contract SugarCoin [

mapping(address => uint256) public balance;

uint256 public totalSupply;

address public treasury;

constructor(address_treasury)[

treasury = _treasury;

]

function mint(address to, uint256 amount) external [

require(msg.sender == treasury, Only treasury);

balance[to] += amount;

totalSupply += amount;

]

function burn(address from, uint256 amount) external [

require(msg.sender == treasury, Only treasury);

require(balance[from]>= amount, Low balance);

balance[from] - = amount;

totalSupply - = amount;

]

function transfer(address to, uint256 amount) external [

require(balance[msg.sender]>= amount, Low balance);

balance[msg.sender] - = amount;

balance[to] += amount;

]

]

Contract C: TraceabilityLedger (asset creation + ownership transfer + audit)

CONTRACT TraceabilityLedger

ENUM Status [CREATED, LISTED, IN_TRANSIT, DELIVERED, PROCESSED, CLOSED]

STRUCT Batch:

id: Bytes32

owner: Address

quantity: Number

unit: String

status: Status

metaCID: String

createdAt: Timestamp

updatedAt: Timestamp

STRUCT TraceEvent:

time: Timestamp

actor: Address

action: String

docCID: String

MAP batches[Bytes32] - > Batch

MAP history[Bytes32] - > List<TraceEvent>

registry: = ParticipantRegistry

FUNCTION createBatch(quantity, unit, metaCID) RETURNS batchId:

registry.requireRole(caller, FARMER)

batchId = HASH(caller, NOW, quantity, metaCID)

batches[batchId] = Batch(batchId, caller, quantity, unit, CREATED, metaCID,

NOW, NOW)

history[batchId].append(TraceEvent(NOW, caller, CREATE, metaCID))

EMIT BatchCreated(batchId)

RETURN batchId

FUNCTION listForSale(batchId, docCID):

REQUIRE batches[batchId].owner == caller

batches[batchId].status = LISTED

batches[batchId].updatedAt = NOW

history[batchId].append(TraceEvent(NOW, caller, LIST, docCID))

EMIT BatchListed(batchId)

FUNCTION transferOwnership(batchId, newOwner, docCID):

REQUIRE batches[batchId].owner == caller

REQUIRE registry.participants[newOwner].active == TRUE

batches[batchId].owner = newOwner

batches[batchId].status = IN_TRANSIT

batches[batchId].updatedAt = NOW

history[batchId].append(TraceEvent(NOW, caller, TRANSFER, docCID))

EMIT OwnershipTransferred(batchId, caller, newOwner)

FUNCTION confirmDelivery(batchId, docCID):

REQUIRE batches[batchId].owner == caller

batches[batchId].status = DELIVERED

batches[batchId].updatedAt = NOW

history[batchId].append(TraceEvent(NOW, caller, DELIVER, docCID))

EMIT DeliveryConfirmed(batchId)

FUNCTION markProcessed(inputBatchId, outputQty, outputUnit, docCID) RETURNS outputBatchId:

registry.requireRole(caller, MILL)

REQUIRE batches[inputBatchId].owner == caller

batches[inputBatchId].status = PROCESSED

batches[inputBatchId].updatedAt = NOW

history[inputBatchId].append(TraceEvent(NOW, caller, PROCESS_INPUT, docCID))

outputBatchId = HASH(inputBatchId, NOW, outputQty, docCID)

batches[outputBatchId] = Batch(outputBatchId, caller, outputQty, outputUnit,

PROCESSED, docCID, NOW, NOW)

history[outputBatchId].append(TraceEvent(NOW, caller, PROCESS_OUTPUT, docCID))

EMIT BatchProcessed(inputBatchId, outputBatchId)

RETURN outputBatchId

FUNCTION getTrace(batchId) RETURNS List<TraceEvent > :

RETURN history[batchId]

Contract D: TradeEscrow (trade execution + payment settlement)

interface ISugarCoin [

function transferFrom(address from, address to, uint256 amount) external;

function transfer(address to, uint256 amount) external;

]

contract TradeEscrow [

enum State [INIT, PAID, SETTLED, CANCELED]

struct Trade [

address seller;

address buyer;

uint256 price;

State state;

]

mapping(bytes32 => Trade) public trades;

ISugarCoin public token;

constructor(address _token) [

token = ISugarCoin(_token);

]

function initiateTrade(bytes32 tradeId, address buyer, uint256 price)

external [

trades[tradeId] = Trade(msg.sender, buyer, price, State.INIT);

]

function deposit(bytes32 tradeId) external [

Trade storage t = trades[tradeId];

require(msg.sender == t.buyer && t.state == State.INIT, Not allowed);

token.transferFrom(msg.sender, address(this), t.price);

t.state = State.PAID;

]

function settle(bytes32 tradeId) external [

Trade storage t = trades[tradeId];

require(msg.sender == t.buyer && t.state == State.PAID, Not allowed);

token.transfer(t.seller, t.price);

t.state = State.SETTLED;

]

function cancel(bytes32 tradeId) external

SC ↔ Fiat conversion

Most papers implement fiat conversion via a regulated off-chain payment gateway and record only verifiable mint_burn actions on-chain.

Off-chain component: FiatGateway

INPUT: user requests SC purchase or redemption

CASE 1: Fiat → SC

- verify fiat deposit via banking rails

- compute amountSC using fixed_variable rate policy

- call SugarCoin.mint(user, amountSC)

- store receipt_encrypted details in IPFS → receiptCID

- emit on-chain event FiatDeposited(user, amountSC, receiptCID)

CASE 2: SC → Fiat

- user sends SC to Treasury OR approves burn

- gateway validates redemption request

- call SugarCoin.burn(user, amountSC)

- initiate fiat payout via banking rails

- store payout proof in IPFS → payoutCID

- emit on-chain event FiatRedeemed(user, amountSC, payoutCID)

**Algorithm 1. On-chain traceability and trade settlement (Smart contract workflow)**

Input: ActorRequests (registration, batch creation, trade, transfer), encrypted

metadata pointers CID (IPFS), priceSC

Output: Immutable trace log History[batchId], settled trade state, updated

ownership and balances

1. Participant onboarding

2. If request.type = REGISTER then

3. Require caller = Regulator_Admin

4. Store (participantAddress, role, active = true, kycCID) in ParticipantRegistry

5. Emit ParticipantRegistered(participantAddress, role)

6. End if

7. Batch creation (Farm)

8. If request.type = CREATE_BATCH then

9. Require caller.role = FARMER and caller.active = true

10. Compute batchId ← Hash(caller, timestamp, quantity, metaCID)

11. Store Batch(batchId, owner = caller, quantity, unit, status = CREATED, metaCID)

12. Append TraceEvent(time, caller, CREATE, metaCID) to History[batchId]

13. Emit BatchCreated(batchId)

14. End if

15. Trade execution (Escrow-based)

16. If request.type = INITIATE_TRADE then

17. Require caller = currentOwner(batchId)

18. Create tradeId ← Hash(batchId, seller = caller, buyer, timestamp)

19. Store Trade(tradeId, batchId, seller, buyer, priceSC, state = INIT)

20. Emit TradeInitiated(tradeId)

21. End if

22. Payment escrow

23. If request.type = DEPOSIT_SC then

24. Require caller = buyer(tradeId) and Trade.state = INIT

25. Transfer priceSC from buyer to EscrowContract (via transferFrom)

26. Set Trade.state ← PAID

27. Emit SCPaymentLocked(tradeId)

28. End if

29. Ownership transfer _ shipment

30. If request.type = TRANSFER_OWNERSHIP then

31. Require caller = seller(tradeId) and Trade.state = PAID

32. Set Batch.owner ← buyer(tradeId) and Batch.status ← IN_TRANSIT

33. Append TraceEvent(time, caller, TRANSFER, docCID) to History[batchId]

34. Emit OwnershipTransferred(batchId, seller, buyer)

35. End if

36. Delivery confirmation + settlement

37. If request.type = CONFIRM_DELIVERY then

38. Require caller = buyer(tradeId) and Trade.state = PAID

39. Set Batch.status ← DELIVERED

40. Append TraceEvent(time, caller, DELIVER, docCID) to History[batchId]

41. Release escrow: transfer priceSC from EscrowContract to seller

42. Set Trade.state ← SETTLED

43. Emit PaymentReleased(tradeId)

44. End if

45. Processing (Mill)

46. If request.type = PROCESS_BATCH then

47. Require caller.role = MILL and caller = Batch.owner

48. Set Batch.status ← PROCESSED

49. Create outputBatchId ← Hash(inputBatchId, timestamp, outputQty, outputCID)

50. Store output Batch(outputBatchId, owner = caller, outputQty, unit,

status = PROCESSED, outputCID)

51. Append TraceEvent(time, caller, PROCESS_INPUT, inputDocCID) to History[inputBatchId]

52. Append TraceEvent(time, caller, PROCESS_OUTPUT, outputDocCID) to History[outputBatchId]

53. Emit BatchProcessed(inputBatchId, outputBatchId)

54. End if

55. Audit _ trace query

56. If request.type = TRACE_QUERY then

57. Return History[batchId] (ordered list of TraceEvents)

58. End if

**Algorithm 2. SC–Fiat conversion with on-chain audit events**

Input: User conversion request (fiat→SC or SC→fiat), conversion rate policy R, banking confirmation, IPFS evidence CIDs

Output: Mint_Burn actions on-chain, auditable events (FiatDeposited, FiatRedeemed), updated balances

1. Receive conversion request from user u with direction dir ∈ [FIAT_TO_SC, SC_TO_FIAT]

2. If dir = FIAT_TO_SC then

3. Collect fiat deposit using regulated payment rails

4. Verify deposit confirmation (bankTxConfirmed = true)

5. Compute amountSC ← convert(fiatAmount, R)

6. Store encrypted receipt_proof in IPFS → receiptCID

7. Call on-chain SugarCoin.mint(u, amountSC) from Treasury_Gateway

8. Emit FiatDeposited(u, fiatAmount, amountSC, receiptCID)

9. End if

10. If dir = SC_TO_FIAT then

11. User approves burn or transfers amountSC to Treasury address

12. Verify request (AML_KYC checks, balance availability)

13. Store encrypted payout proof in IPFS → payoutCID

14. Call on-chain SugarCoin.burn(u, amountSC) from Treasury_Gateway

15. Initiate fiat payout using regulated payment rails

16. Emit FiatRedeemed(u, amountSC, fiatAmount, payoutCID)

17. End if

18. Auditability

19. Any auditor_regulator can query blockchain events

20. FiatDeposited and FiatRedeemed events link to IPFS CIDs for verification evidence

IPFS storage, encryption, and access control Sensitive stakeholder data (e.g., farmer identity attributes, contracts, invoices, and compliance documents) are not stored directly on-chain. Instead, the system stores encrypted payloads in IPFS and records only content identifiers (CIDs) on-chain. Each record uses hybrid encryption to provide confidentiality, integrity, and controlled sharing.

Data encryption (at rest) For each private record D: 1. A fresh symmetric Data Encryption Key (DEK) is generated per record: DEK ← Random(256 bits). 2. The payload is encrypted using AES-256-GCM: C ← AES-256-GCM-Encrypt(DEK, D, AAD), where AAD (associated authenticated data) includes the record type, batchId, timestamps, and issuer address to bind ciphertext integrity to the supply-chain context. 3. A cryptographic hash of ciphertext is computed for additional auditability: h ← SHA-256(C) (optional if using IPFS CID only). The resulting ciphertext C is uploaded to IPFS, producing a CID CIDdata. Only CIDdata is stored on-chain in the trace log.

Key wrapping and authorized sharing (key management) To allow only specific parties to decrypt D, the DEK is wrapped (encrypted) for each authorized recipient using their public key. The envelope (or its CID) is referenced on-chain. This approach ensures that compromise of one recipient key does not expose other records because DEKs are per-record and independent.

Data access control and authorization Access control is enforced at two layers: 1. On-chain role and permission checks: A ParticipantRegistry contract maintains roles (e.g., Farmer, Mill, Distributor, Retailer, Regulator). Smart contract functions that write CIDs (create batch, transfer, process) require an active role. In addition, read access to sensitive CIDs can be restricted to authorized roles (depending on public vs permissioned design). 2. Cryptographic access control (decryption gate): Even if a CID is visible, decryption requires the recipient’s private key to unwrap and obtain. Only recipients listed in the envelope can decrypt the payload. The decryption workflow is: o Retrieve (or envelope) from IPFS. o Verify authorization against on-chain policy (optional). o Use private key to decrypt. o Decrypt ciphertext.

Transport security and integrity • All client ↔ gateway communication uses TLS 1.2 + . • Integrity is ensured by IPFS content addressing (CID) and AES-GCM authentication tags; optional SHA-256 hash h is used for independent verification and audit logging.

## Experiments and results

This section contains simulations, real-time use cases implementation of our proposed framework, and performance results. The following are some assumptions:

- Miners or groups of miners in the system shall not have fifty-one percent hash power
- Only registered users would be able to buy and sell products

**Fig 10:**
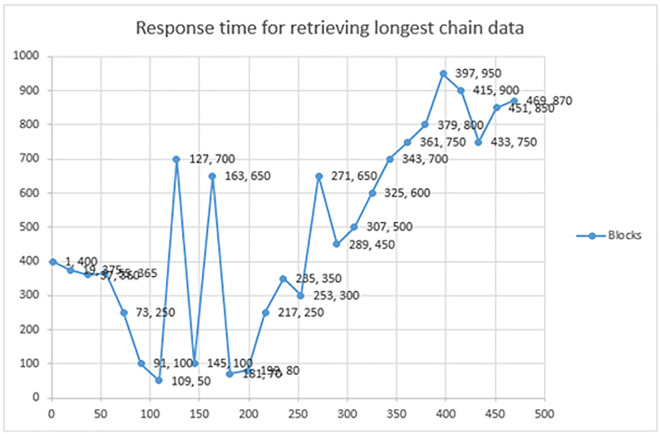
Response time for retrieving longest chain data.

For concluding experiments, we have used a Linux-based VPS server with 16 cores, 32 GB RAM, and 1 TB SSD. Bandwidth was 40 mbits/sec. The Testnet Smart Chain blockchain (PoS) is used and interacted with it through the web3.0 open source wallet MetaMask for deployment of our framework. The Remix Integrated Development Environment (IDE) has been used for smart contract writing. Postman was used for HTTP request interaction with blockchain. The ValidateBlock and AccessChain functions were used to analyze the performance of the blockchain. A similar procedure was used for other performance evaluations. A customized Python script was used to send fifty thousand transactions to the network with the help of the Web3.0 Python library. The fifty thousand transactions dataset was uploaded and saved on Github [28]. The dataset illustrates 4 details of transactions which are Transaction count, Transaction sender, transaction data, and signer private key. The transaction count is an index of transactions. The transaction sender was the public address of the transaction originator. The Transaction data field was sample data inside each transaction. The transaction Signer Private key was a private key that was used to generate the specific public address of the transaction sender. Fig 10 shows the descriptive detail of response time for retrieving the longest chain.

This study deployed five validator nodes on the Binance Smart Chain (BSC) testnet to simulate a consortium blockchain environment. The validator nodes were interconnected using a full-mesh network topology, ensuring direct peer-to-peer communication among all nodes for efficient consensus participation. Each node was configured with identical computational resources to maintain experimental consistency. The test environment was established to evaluate transaction latency, throughput, and consensus performance under controlled conditions. This configuration enables reproducibility of the experimental setup and facilitates comparative analysis in future research. To evaluate the performance and feasibility of the proposed blockchain framework, a controlled experimental environment was established using the Binance Smart Chain (BSC) testnet. The testnet environment was selected to avoid financial costs associated with mainnet deployment while preserving realistic blockchain operational behavior. In this study, five validator nodes were deployed to simulate a consortium blockchain network. Although BSC is typically a public blockchain, the configuration was logically structured to emulate a permissioned consortium setting by restricting validator participation to predefined nodes. Each validator node was responsible for block validation, transaction verification, and participation in the consensus mechanism. The validator nodes were interconnected using a full-mesh topology, meaning that each node maintained direct communication links with every other node in the network. This configuration ensures: • Reduced communication delay during block propagation • Faster consensus agreement • Elimination of single points of failure • Improved network reliability All nodes were provisioned with identical hardware and software configurations to eliminate performance variability caused by infrastructure differences. Uniform resource allocation ensures that experimental results such as latency and throughput are attributable to protocol behavior rather than hardware discrepancies. The experimental framework focused on evaluating the following performance metrics:

1. Transaction Latency – Time taken from transaction submission to block confirmation.
2. Throughput (TPS) – Number of transactions processed per second.
3. Consensus Performance – Block finality time and validation efficiency.
4. Network Stability – Behavior under varying transaction loads.

Controlled workloads were generated to simulate real-world consortium usage scenarios. The setup enables reproducibility by clearly defining node count, topology, resource allocation, and evaluation parameters, allowing future researchers to replicate or extend the experiments under comparable conditions.

These transactions were used as samples for our data analysis. Five experiments and comparisons have been performed for the proposed framework evaluation with respect to other available solutions as follows:

i. Response time calculation for retrieving longest chain data in milliseconds for the proposed framework
ii. New blocks addition latency in blockchain and other statistics comparison
iii. Transactions, blocks, and data addition latency and blocks confirmations comparisons
iv. Average transaction fee comparison of the proposed framework and other solutions
v. Overall performance evaluation comparison with multiple blockchain solutions

To retrieve blockchain data AccessChain function has been used on different blocks. This function helps in retrieving some specific data from multiple blocks across the chain and adds a timestamp to calculate the time used in accessing data. In starting blocks time latency was around 400 ms then it reduced to 91 and then kept increasing over time up to 950 milliseconds. It can be noticed that latency time shows some irrelevant behaviour across multiple blocks which happened due to the decentralized nature of blockchain.

**Fig 11:**
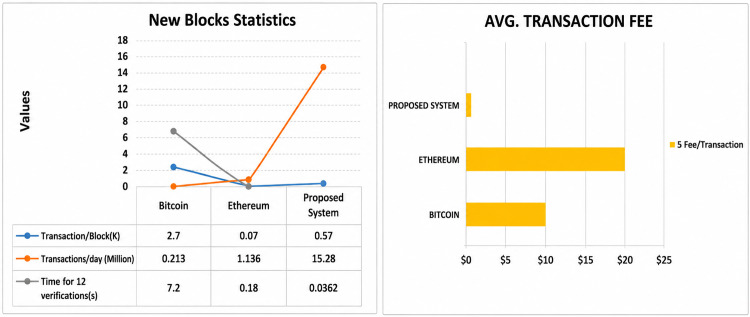
New block statistics (left) and average transaction fee in USD right.

Each time data is retrieved from multiple nodes which causes delay. However, there is a significant increase in retrieving time when block size increases. Response time is very critical for larger DApps where there are multiple users interacting with the blockchain. Response time for our proposed framework is less than 1 second and it is scalable and does not change with more users. Fig 10 shows the response time for retrieving the longest chain with different blocks in pair form. In comparison to other networks, our proposed framework takes very less time to add new blocks to the blockchain. Bitcoin network takes usually 10 minutes and the Ethereum network takes 15 seconds on average to add new blocks to the blockchain. Our proposed framework works with a new block after each 3-second mechanism. New block latency performance is compared in Fig 11. This early block addition makes the proposed system more efficient and can verify new transactions more easily. Due to more delays in blocks Bitcoin and Ethereum networks have daily transaction volumes of 0.213 million and 1.13 million respectively. While proposed framework can add 15.28 million transactions on average to the blockchain each day. Fig 11 (left) shows new block statistics of multiple blockchains and also describes the time required to achieve twelve network confirmations which are necessary for accepting financial payments. The proposed framework can confirm new transactions 6x faster than Ethereum and 200x faster than Bitcoin.

Table 3 explains the Parameters and ICO sale values. Table 4 shows the New Block Transaction. Table 5 Overall comparison with already existing blockchains. Table 6 shows the Comparison of Experiment Results. Table 7 depicts the Estimated adoption cost and effort for non-technical stakeholders. Table 8 Proposed framework comparison with IBM Food and AgriDigital with different dimensions. Table 9 Proposed framework comparison with IBM Food and AgriDigital with different dimensions having Etherium and Blockchain Table 5 explains the overall comparison of the proposed framework with the already existing blockchain. The government can reduce transaction fees up to 0 usd if they afford to introduce their own local blockchain instead of using already existing ones.

**Table 3:** Parameters and ICO sale values.

Parameter	Values
Symbol	SugarCoin (SC)
Type	ERC20
Maximum supply	500 million
Accepted Currency	PKR, Dollar, EUR, BTC etc.
Price	1 SC = 100 USD

**Table 4:** New blocks latency.

Blocks	Latency(milliseconds)
1	400
55	365
109	50
163	650
217	250
271	650
325	600
379	800
433	750
469	870

**Table 5:** Overall comparison with already existing blockchains.

Blockchain	Transaction/Block(k)	Block time(minutes)	Time for 12 verification(ks)	Fee/Transaction	Transactions/day(Million)
Yanez et al. [38]	11	2.8	7.3	$10.2	0.215
Salah et al. [39]	0.071	0.251	0.182	$30	1.14
Nakamoto et. al. [15]	2.7	10	7.2	$10	0.213
Sharma et. al. [16]	0.07	0.25	0.18	$20	1.136
Musamih et. al. [10]	0.07	0.25	0.18	$20	1.136
Proposed System	0.57	0.03	0.0362	$0.30	15.28

**Table 6:** Comparison experiment results.

Dimension	Proposed Sugarcane Framework (This Study)	IBM Food Trust	AgriDigital	Public Ethereum (Baseline)
Primary use-case	Sugarcane farm-to-fork traceability + trading + settlement	Food traceability network across multiple products/supply chains	Digital grain/commodity management; trading + settlement + provenance pilots	General-purpose smart contracts / dApps
Network type	Typically **permissioned/consortium** (recommended for regulated supply chains)	**Permissioned enterprise network**	Enterprise/consortium deployments; pilots reported on private networks	**Public permissionless**
Underlying stack	Ethereum-compatible (smart contracts + tokenization) + IPFS (off-chain)	Built on **Hyperledger Fabric** (enterprise blockchain)	Blockchain-enabled platform; pilots have used **private Ethereum** in early settlement demonstrations	Ethereum mainnet
Consensus	Configurable (depends on deployment: PoA/IBFT/PoS in private settings)	Fabric’s permissioned model (consensus is modular/pluggable depending on deployment)	Not consistently disclosed across deployments; early pilots reported multi-node private Ethereum	PoS (current Ethereum mainnet)
Traceability granularity	Batch/lot-level with on-chain event logs + IPFS metadata	Farm-to-shelf tracking with product history and location visibility)	Commodity lot/title transfer + provenance in pilots (e.g., grain)	Depends on dApp; not domain-specific
Smart contracts / business logic	Explicit smart contracts for: registration, batch creation, transfer, escrow settlement, audit queries	“Smart contract”-like business logic supported in Fabric chaincode model	Supports digitized trade/settlement workflows; blockchain used to establish trust in transactions	Yes; general-purpose
Tokenization / payment	**Native token (Sugar Coin, SC)** for recorded settlement; supports SC↔fiat via gateway	Typically no public token mechanism in the platform’s standard model (enterprise data network)	Focus on commodity settlement and payments (implementation details vary by deployment)	Native ETH; token standards supported
Off-chain storage	**IPFS** for encrypted private data; CID stored on-chain	Enterprise integrations; data shared among authorized participants; storage details vary by implementation	Mix of cloud platform + blockchain components; storage details vary by deployment	External storage optional; not built-in
Privacy / access control	On-chain RBAC + encrypted IPFS payloads + key distribution policy	Permissioned access among known participants; enterprise privacy controls	Permissioned access typical for enterprise commodity workflows (details vary)	Public by default; privacy requires extra tooling
Data transparency for regulators	Strong (audit trail + queryable event logs + optional regulator key access)	Supports rapid traceability and authorized access to supply chain data	Supports transaction auditability in commodity workflows (deployment-dependent)	Public transparency; limited domain semantics
Public performance benchmarking availability	Yes (you can report block latency, throughput, gas cost, verification time)	Not generally benchmarked publicly at node/latency level (production configs are proprietary)	Not consistently benchmarked publicly across deployments	Public metrics available, but not comparable to private/permissioned setups

**Table 7:** Estimated adoption cost and effort for non-technical stakeholders.

Component	Description	Estimated Cost / Effort	Notes
User device	Smartphone or basic computer with internet access	Low (existing devices in most cases)	No specialized hardware required
Network connectivity	Mobile data or broadband access	Low–Moderate	Dependent on rural infrastructure availability
Software access	Web/mobile application interface	Low	Browser-based or lightweight mobile app
User onboarding	Account creation, identity verification	Low (1–2 hours per user)	Can be supported by cooperatives or agents
Training requirement	Task-oriented training (batch entry, confirmation, payments)	Low (half-day session)	No blockchain knowledge required
Key management	Abstracted via application or custodial service	Low	Reduces cryptographic complexity for users
Transaction fees	Smart contract execution costs	Low–Moderate	Optimized via batching and permissioned networks
Maintenance effort	Periodic updates and support	Low	Managed centrally by platform operator
Regulatory compliance overhead	Reporting and audit access	Low–Moderate	Mostly automated via smart contract logs

**Table 8:** Proposed framework comparison with IBM Food and AgriDigital with different dimensions.

Dimension	Proposed Sugarcane Framework (This Study)	IBM Food Trust	AgriDigital	Public Ethereum (Baseline)
Primary use-case	Sugarcane farm-to-fork traceability + trading + settlement	Food traceability network across multiple products/supply chains	Digital grain/commodity management; trading + settlement + provenance pilots	General-purpose smart contracts / dApps
Network type	Typically **permissioned/consortium** (recommended for regulated supply chains)	**Permissioned enterprise network**	Enterprise/consortium deployments; pilots reported on private networks	**Public permissionless**
Underlying stack	Ethereum-compatible (smart contracts + tokenization) + IPFS (off-chain)	Built on **Hyperledger Fabric** (enterprise blockchain)	Blockchain-enabled platform; pilots have used **private Ethereum** in early settlement demonstrations	Ethereum mainnet
Consensus	Configurable (depends on deployment: PoA/IBFT/PoS in private settings)	Fabric’s permissioned model (consensus is modular/pluggable depending on deployment)	Not consistently disclosed across deployments; early pilots reported multi-node private Ethereum	PoS (current Ethereum mainnet)
Traceability granularity	Batch/lot-level with on-chain event logs + IPFS metadata	Farm-to-shelf tracking with product history and location visibility	Commodity lot/title transfer + provenance in pilots (e.g., grain)	Depends on dApp; not domain-specific
Smart contracts / business logic	Explicit smart contracts for: registration, batch creation, transfer, escrow settlement, audit queries	“Smart contract”-like business logic supported in Fabric chaincode model	Supports digitized trade/settlement workflows; blockchain used to establish trust in transactions	Yes; general-purpose
Tokenization / payment	**Native token (Sugar Coin, SC)** for recorded settlement; supports SC↔fiat via gateway	Typically no public token mechanism in the platform’s standard model (enterprise data network)	Focus on commodity settlement and payments (implementation details vary by deployment)	Native ETH; token standards supported
Off-chain storage	**IPFS** for encrypted private data; CID stored on-chain	Enterprise integrations; data shared among authorized participants; storage details vary by implementation	Mix of cloud platform + blockchain components; storage details vary by deployment	External storage optional; not built-in
Privacy / access control	On-chain RBAC + encrypted IPFS payloads + key distribution policy	Permissioned access among known participants; enterprise privacy controls	Permissioned access typical for enterprise commodity workflows (details vary)	Public by default; privacy requires extra tooling
Data transparency for regulators	Strong (audit trail + queryable event logs + optional regulator key access)	Supports rapid traceability and authorized access to supply chain data	Supports transaction auditability in commodity workflows (deployment-dependent)	Public transparency; limited domain semantics
Public performance benchmarking availability	Yes (you can report block latency, throughput, gas cost, verification time)	Not generally benchmarked publicly at node/latency level (production configs are proprietary)	Not consistently benchmarked publicly across deployments	Public metrics available, but not comparable to private/permissioned setups

**Table 9:** Proposed framework comparison with IBM Food and AgriDigital with different dimensions having Etherium and Blockchain.

Dimension	Proposed Sugarcane Framework (This study)	IBM Food Trust	AgriDigital	Ethereum (baseline)	Bitcoin (baseline)
Primary domain	Sugarcane traceability + trading/settlement	Food traceability network (multi-product)	Commodity trading/settlement (grains) + provenance pilots	General-purpose smart contract platform	Cryptocurrency payment network
Network model	Consortium/permissioned (recommended for regulated supply chains)	Permissioned enterprise network	Enterprise deployments; pilots reported on private multi-node Ethereum	Public permissionless	Public permissionless
Core technology	Smart contracts + token + IPFS (off-chain encrypted data)	Built on Hyperledger Fabric / IBM Blockchain platform	Blockchain used for settlement/provenance (pilot on private Ethereum)	EVM smart contracts	UTXO scripts (limited programmability)
Traceability granularity	Batch/lot-level provenance with on-chain events + IPFS CIDs	Item/lot traceability; query by product identifiers (e.g., GTIN/UPC)	Lot/title-transfer focus + provenance in pilots	Depends on dApp (not domain-specific)	Not designed for supply-chain traceability
Smart contract / chaincode logic	Explicit workflow functions: registration, batch create, transfer, escrow settle, trace query	Fabric chaincode + enterprise access controls	Workflow depends on deployment; settlement pilots documented	Yes	No (limited scripting)
Privacy & access control	RBAC + encrypted off-chain records (IPFS) + key distribution	Permissioned sharing; data owners control access	Enterprise access controls (implementation	Public-by-default; privacy needs add-ons	Public-by-default
Token/payment model	Sugar Coin (SC) for settlement + escrow; SC↔fiat via gateway	Typically no native cryptocurrency reliance	Focus on settlement of agricultural commodities; pilots executed blockchain settlement	ETH and ERC-20 tokens	BTC
Benchmark feasibility vs your experiments	**Direct benchmarking possible** (your controlled testbed)	Direct low-level benchmarking usually not feasible publicly (production configs not exposed)	Direct benchmarking varies; pilots described but production telemetry limited	Direct benchmarking possible	Direct benchmarking possible

## Discussion

Tests show that the system we developed can process blocks in about 3 seconds. That’s much quicker than Bitcoin, which takes 10 minutes, and even faster than Ethereum at 15 seconds. This speed really meets what’s needed for keeping track of things in real-time within supply chains. Now, when you look at centralized options like IBM Food Trust, they often use a setup involving a group of organizations, which can be expensive and might leave smaller farmers out. Our approach is different because it’s decentralized. We use the affordable Binance Smart Chain and a special token called Sugar Coin to make it much easier and less costly for everyone to join in. Plus comparing it to something like AgriDigital that mostly handles settling transactions, our system does both settling (using Sugar Coin) and tracking products (using IPFS and smart contracts) all together in one seamless package. One of the main hurdles we’ve noticed during implementation is how much prices tend to swing. To help with this, we’re suggesting the Sugar Coin as a utility token within the system. Down the road, we might look into linking SC to a stablecoin reserve, which could guarantee farmers steady pricing even when the crypto market is unpredictable.

Research Methodology This study adopts a constructive research approach, designing a technical artifact (the blockchain framework) to solve the specific domain problems of the sugarcane industry. Smart Contract Implementation The core business logic is implemented using Solidity smart contracts on the Binance Smart Chain (BSC). BSC was selected over Ethereum for its lower transaction fees and higher throughput. The contracts govern the issuance of Sugar Coin (SC) and the tracking of product batches.

**Algorithm Product Registration**

Function RegisterProduct(UserID, ProductDetails, IPFS_Hash):
If User is Authorized:
Create new ProductStruct
Assign UniqueID = GenerateHash(Timestamp, UserID)
Store ProductStruct on Ledger
Emit Event(ProductRegistered)
Else: Revert Transaction

Data Privacy and Encryption To address the privacy concerns of farmers and retailers, sensitive data (such as exact harvest location and personal financial details) is not stored directly on the public ledger. Instead, we utilize the Inter-Planetary File System (IPFS). • Encryption: Before uploading to IPFS, data is encrypted using the AES-256 standard. • Key Management: The decryption keys are shared only between authorized parties (e.g., the farmer and the specific retailer) via an off-chain secure channel, ensuring that while the hash of the data is immutable on the blockchain, the content remains private.

Sugar Coin (SC) pricing and stabilization mechanism Sugar Coin (SC) is designed as a stable-value settlement token for sugarcane supply-chain transactions rather than a freely floating cryptocurrency. While an indicative exchange rate of 1 SC = 100 USD is defined for accounting and settlement purposes, price stability is maintained through a policy-driven stabilization framework. SC issuance and redemption are governed by a controlled mint–burn mechanism, operated through a regulated treasury or gateway entity. Under this model, SC tokens are minted only when an equivalent fiat value is deposited through authorized payment channels, and burned when users redeem SC for fiat currency. This approach aligns SC with a fiat-backed stable token design, minimizing exposure to speculative volatility. To address demand fluctuations, the system supports threshold-based supply adjustment policies, where temporary excess demand or surplus triggers controlled minting or burning within predefined limits. These mechanisms ensure that SC retains a stable settlement value while supporting predictable pricing for farmers, mills, and other supply-chain participants.

Government supervision and regulatory compliance Government supervision within the proposed framework is implemented through explicit on-chain regulatory controls rather than off-chain monitoring alone. Authorized regulatory bodies are registered as privileged participants with role-based access control (RBAC), enabling them to audit transactions, monitor token circulation, and enforce compliance policies. Regulatory oversight is supported by compliance-aware smart contracts that generate immutable event logs for critical operations such as token issuance, batch transfers, settlement, and redemption. Sensitive off-chain records stored in IPFS are encrypted and shared using controlled key distribution, where regulatory authorities may be included as authorized recipients for lawful inspection. This design enables transparent auditing, prevents unauthorized trading or hoarding, and supports policy enforcement (e.g., transaction suspension or investigation) while preserving participant privacy.

**Algorithm 3. SC price stabilization and regulatory flow Input: User request (SC buy, SC sell, trade settlement), fiat confirmation, compliance rules Output: Stable SC circulation, auditable regulatory records**

1. SC issuance (Fiat → SC)

2. User submits fiat deposit through an authorized gateway

3. Gateway verifies fiat settlement via regulated banking rails

4. Compute amountSC = fiatAmount / 100

5. Call SugarCoin.mint(user, amountSC)

6. Emit SCMinted(user, amountSC, timestamp)

7. Record encrypted receipt in IPFS → receiptCID

8. SC redemption (SC → Fiat)

9. User submits redemption request

10. Verify user balance and compliance rules

11. Call SugarCoin.burn(user, amountSC)

12. Initiate fiat payout via gateway

13. Emit SCRedeemed(user, amountSC, timestamp)

14. Store encrypted payout proof in IPFS → payoutCID

15. Regulatory supervision

16. For each mint, burn, or trade event:

17. Log immutable event on-chain

18. Allow regulator nodes to query events and trace batches

19. If anomaly detected (e.g., hoarding, abnormal volume), flag transaction for review

20. Audit and enforcement

21. Regulators retrieve encrypted records using authorized decryption keys

22. Compliance actions (monitoring, suspension, reporting) are executed as per policy

**Adoption by non-technical users and operational feasibility** Adoption by non-technical stakeholders, including farmers and small- and medium-sized enterprises (SMEs), is a critical factor for the practical deployment of the proposed framework. To minimize technical barriers, the system is designed to operate through simple mobile or web-based interfaces, abstracting blockchain-specific operations such as key management, transaction signing, and gas handling. Users interact with familiar workflows (e.g., batch registration, delivery confirmation, payment receipt) without direct exposure to underlying blockchain mechanisms. Training requirements are expected to be modest and task-oriented. Basic onboarding sessions can focus on application usage rather than blockchain concepts, reducing training time and associated costs. In practice, training can be delivered through cooperatives, agricultural extension services, or local industry associations. Equipment requirements are intentionally kept minimal, relying on widely available smartphones or basic computing devices with internet connectivity, rather than specialized hardware. This design choice supports scalability in rural and resource-constrained environments.

**Cross-border transactions and regulatory readiness** Although the primary focus of this study is domestic sugarcane supply-chain management, the proposed framework is designed to be extensible to international trade scenarios. Cross-border deployment introduces additional considerations, including tariffs, exchange-rate variability, customs regulations, and jurisdiction-specific compliance requirements. To accommodate these factors, the framework supports modular policy layers that can integrate tariff schedules, tax rules, and customs documentation as metadata linked to transactions. Exchange-rate variability can be managed through the use of stable-value settlement mechanisms, where Sugar Coin functions as an intermediate settlement unit while fiat conversion occurs through regulated gateways at prevailing rates. Compliance with international trade regulations can be supported by role-based regulatory access, auditable transaction logs, and integration with existing customs and trade reporting systems. While full implementation of cross-border functionality is beyond the scope of this study, these design considerations ensure that the framework is aligned with practical requirements for future international adoption.

## Conclusion

A blockchain-based framework for the sugarcane supply chain is proposed in this research for the traceability and price control of sugarcane products. The framework has been deployed on the Binance Smart Chain (BSC) network, which is faster and consumes fewer transaction fees than existing blockchains such as Bitcoin and Ethereum. The proposed solution uses a smart contract-based token named Sugar Coin (SC) for purchasing various supply chain steps. The SC ensures that farmers receive fair wages for their efforts, and all other stakeholders receive good profits. The proposed framework also helps to regulate the export of sugar, thus saving the government effort and money by avoiding the import of expensive sugar in off-seasons. To increase data security, availability, and stakeholder trust, the InterPlanetary File System (IPFS) is used for storing encrypted data of farmers, companies, and retailers. The potential future directions of this work include customizing smart contracts to make them more optimized in performance and reduce transaction costs by optimizing transactional functions. Dedicated smart wallets for managing Sugar Coin and mobile traceability apps are also part of future work. The dedicated wallet allows users to update and view their off-chain IPFS data. The current system is based on blockchain, which may be difficult for illiterate farmers to understand. However, introducing a user-friendly interactive mobile application with images, sound, and guidance can help stakeholders understand and use the system more easily. In this paper, we have only covered national (Pakistan) level trading, while trading across International borders was out of scope. The transaction fee can be reduced further with the help and support of governments. For International fiat currency exchanges some changes in the economic model will be required.

This study presents a blockchain-based framework for sugarcane supply chain management that utilises smart contracts and IPFS storage to enhance transparency, traceability, and pricing equity, resulting in superior cost efficiency and performance compared to existing solutions. Key refinements include the automation of fair farmer compensation and the provision of verifiable quality metrics for retailers. However, the model is currently limited by its focus on the Pakistani domestic market, excluding cross-border complexities such as international taxation and currency volatility, as well as the digital literacy barriers faced by farmers. To address these constraints, future work will focus on developing icon-driven mobile interfaces for non-tech-savvy users, implementing price stabilization protocols for the Sugar Coin, and conducting real-world pilot testing with local sugar mills to validate practical scalability.

## Supporting information

S1 Data Data set. (CSV)
