# Supplementary material for: Enhancing traceability and trust in the sugarcane supply chain using a blockchain-based framework with smart contracts and tokenization
Source: PLoS One. 2026 Aug 4;21(8):e0352021. doi: 10.1371/journal.pone.0352021 (PMC13436780; doi:10.1371/journal.pone.0352021)
Supplement: Supplementary file 1 [file pone.0352021.s003.docx]

**Submission ID**:

Enhancing Traceability and Trust in the Sugarcane Supply Chain Using a Blockchain-Based Framework with Smart Contracts and Tokenization

**To:** PLOS ONE

Dear Editor,

First, we are extremely grateful to the editor and all the reviewers who conducted a rigorous and constructive review of our article . Certainly, all the review points were very well directed, and we managed to improve our manuscript a great deal and had to work a lot to address all the changes suggested by the reviewers.

We have incorporated all the suggested reviews and comments provided by the reviewers and made a careful revision of the manuscript. We have tried our level best to improve this manuscript by organizing and adding more references to the available relevant literature, and by presenting the material more constructively and effectively. This has certainly raised the quality of this research work and will make it a valuable contribution to the community. We hope that we have managed to make the required changes suggested by the valuable reviewers.

Please see our responses in line with the editor’s and reviewers’ concerns in green color.

Best regards,

Farooq et al.

Reviewer #1

**Reviewer#1, Concern # 1:** The abstract is too long, especially its introductory part. This section should be shortened and focused on presenting the paper’s contributions, novelty, and implications..

**Author response:** Thank you for your valuable comment. As per the reviewer's suggestion, we have updated the Abstract and change is made in the updated manuscript, presenting the paper’s contributions, novelty, and implications.

**Author action:** We have updated and revised the Abstract section of the manuscript.

**Reviewer#1, Concern # 2:** The writing style of the introduction resembles a literature review and needs to be rewritten. Many of these parts can be included under the Literature Review section and combined with the Related Work.

**Author response:** Thank you for your valuable comment. According to the reviewer's suggestion, we have already updated the introductions section and have reflected the contributions, novelty, and implications. Secondly added the different specific comparison Table 1 in the introduction section. We have added the main contribution part in the manuscript in the introduction section and differentiated it from related work.

**Author action:** We have revised and updated Table 1 in the manuscript and the introduction section.

**Reviewer#1, Concern # 3:** Add a Methodology section to the paper. The research methodology requires explanation and clarification

**Author response:** Thank you for your valuable comment. As per the reviewer's suggestion, we have highlighted and added the methodology section details in the Materials and Methods section. As the PlosOne template has no separate section, we have added the details in it. For reference, pages 16 to 22 in the updated manuscript.

**Author action:** We have revised and updated the Materials and Methods section of the updated manuscript.

**Reviewer#1, Concern # 4:** Add a Discussion section and compare your findings with those of similar studies.

**Author response:** Thank you for your valuable comment. As per the reviewer's suggestion, we have added the discussion section under the Experiments and Results section. The comparison and other dimension comparisons tables are made and mentioned in this section. For reference, see Table No 6, 7, 8 and 9.

**Author action:** We have revised and updated the Experiments and Results section of the manuscript.

**Reviewer#1, Concern # 5:** Rewrite the Conclusion section. It should include research limitations and future recommendations, organized under a separate subsection.

**Author response:** Thank You for the valuable comment. We have updated the conclusion section and mentioned the limitations and future directions in the manuscript with alignment as well.

**Author action:** We updated the manuscript by reviewing and rewriting the conclusion in the updated manuscript.

**Reviewer#1, Concern # 6:** Provide a detailed statement of the paper’s contributions and implications.

**Author response:** We appreciate the reviewer's keen observation article contribution and implications for this we have updated the instruction and discussion section in the updated manuscript.

**Author action:** We updated the manuscript, and updated as per reviewers’ suggestion. The changes done in updated manuscript.

**Reviewer#1, Concern # 7:** You may prepare a table for the Literature Review section and summarize some relevant studies along with brief critical remarks.

**Author response:** We appreciate the reviewer's keen observation regarding the literature review and have updated the Introduction section accordingly. Tables 1 and 2 are made for this change. As per the suggestion, updated in the manuscript.

**Author action:** We updated the manuscript by reviewing and making the new tables for the comparison with existing studies. Updated the manuscript.

Reviewer #2:

**Reviewer#2, Concern # 1:** Methodology Section Needs Further Detailing:

Specific code or pseudocode for smart contracts is not presented. It is recommended to supplement with the implementation logic of key functions or flowcharts.

The IPFS storage mechanism and data encryption methods are described too broadly. It is recommended to specify the encryption algorithms, key management strategies, and data access control mechanisms.

**Author response:** Thank you for your valuable comment. As per the reviewer's suggestion, we have highlighted and update the Materials and Method section for the details of methodology. The Specific code and algorithms are added of smart contract and how it deals. IPFS storage mechanism and data encryption section mentioned in the materials and methods and discussion sections of the updated manuscript.

**Author action:** We have updated and revised the manuscript by modifying the Materials and Methods section and discussion section in the updated manuscript.

**Reviewer#2, Concern # 2:** Experimental Comparison Could Be More Comprehensive:

Currently, comparisons are only made with a few blockchain systems (e.g., Bitcoin, Ethereum). It is recommended to include comparative analysis with recent agricultural blockchain projects (e.g., AgriDigital, IBM Food Trust).

Details such as the number of nodes and network topology are not mentioned in the experimental environment. It is recommended to supplement these for enhanced reproducibility.

**Author response:** Thank you for your valuable and much-appreciated comments. The reviewer's valuable input is clearly mentioned under the Experiments and Results section and in the discussion section as well. New Tables 6, 7, 8, and 9 are introduced to present a comparative analysis of recent agricultural blockchain studies, highlighting the impact of different network topologies and environmental factors on experimental outcomes.

**Author action:** We have updated and revised the manuscript by modifying the Materials and Methods section and the discussion section in the updated manuscript.

**Reviewer#2, Concern # 3:** Economic Model and Policy Feasibility:

The pricing mechanism for Sugar Coin (1 SC = 100 USD) lacks strategies for dealing with market fluctuations. It is recommended to discuss price stabilisation mechanisms (e.g., pegged reserves, algorithmic stablecoins).

The text mentions "government-supervised transactions" but does not specify the regulatory mechanisms and compliance designs. It is recommended to supplement this.

**Author response:** We appreciate the reviewer’s insightful suggestion. To provide a more specific, detailed Economic Model and Policy Feasibility, we have incorporated under the Material and Method section a proposed framework description of Algorithm 3, and for regulatory mechanisms and compliance designs mentioned the algorithm of on – chain traceability and trade settlement under the Material and Method section.

**Author action:** To address the reviewer's comment, we have updated the manuscript by providing details as mentioned.

**Reviewer#2, Concern # 4:** User Adoption and Implementation Barriers:

The acceptance by non-technical users such as farmers and SMEs, training costs, and equipment requirements are not discussed sufficiently.

International cross-border transactions are mentioned only as future work. However, issues like tariffs, exchange rates, and compliance need to be considered in advance for practical application.**.**

**Author response:** Thank you for your valuable suggestion. We have included the discussion section, and all implementation barriers are discussed in this section, including non-technical users, cross border transactions and practical applications.

**Author action:** We updated the manuscript by adding discussion section in the updated manuscript.

**Reviewer#2, Concern # 5:** Literature Review Could Be More Systematic:

The review of related work on blockchain applications in agricultural supply chains could be further structured by classifying and summarizing based on technology type (traceability, payments, contracts, etc.) or crop type..

**Author response:** Thank you for the insightful comment. We have updated the literature review sections and added the comparison tables as well.

**Author action:** We have modified and updated the Related work section in the updated manuscript.

**Reviewer#2, Concern # 6:** Formatting and Expression Details:

Some figure and table numbers and their references are inconsistent (e.g., mentions of Fig. 3, Fig. 4, etc., in the text but not fully displayed in the submitted content).

Reference formatting needs uniformity (e.g., author name abbreviations, journal name abbreviations).

**Author response:** We thank the reviewer for highlighting these issues. All figure and table numbers have now been carefully reviewed and corrected to ensure consistency between in-text citations and the displayed content. Missing or incorrectly referenced figures (e.g., Fig. 3, Fig. 4) have been properly added, renumbered, or cross-verified throughout the manuscript. Additionally, the reference list has been revised to ensure uniform formatting. Author name abbreviations and journal title abbreviations have been standardized in accordance with the journal’s guidelines. These corrections improve clarity, consistency, and overall manuscript quality.

**Author action:** We updated the manuscript by revised the manuscript for this complete change.

**Reviewer#2, Concern # 7:** PLOS authors have the option to publish the peer review history of their article (what does this mean?). If published, this will include your full peer review and any attached files..

**Author response:** Thank you for your insightful comment. In response to your feedback, yes, we agree to publish the peer review history of our article. We understand that this will include the full peer review reports, author responses, and any attached files. We support transparency in the peer review
