# Supplementary material for: Enhancing traceability and trust in the sugarcane supply chain using a blockchain-based framework with smart contracts and tokenization
Source: PLoS One. 2026 Aug 4;21(8):e0352021. doi: 10.1371/journal.pone.0352021 (PMC13436780; doi:10.1371/journal.pone.0352021)
Supplement: Supplementary file 2 [file pone.0352021.s004.docx]

**Submission ID**: PONE-D-25-25482R1

Enhancing Traceability and Trust in the Sugarcane Supply Chain Using a Blockchain-Based Framework with Smart Contracts and Tokenization

**To:** PLOS ONE

Dear Editor,

First, we are extremely grateful to the editor and all the reviewers who conducted a rigorous and constructive review of our article . Certainly, all the review points were very well directed, and we managed to improve our manuscript a great deal and had to work a lot to address all the changes suggested by the reviewers.

We have incorporated all the suggested reviews and comments provided by the reviewers and made a careful revision of the manuscript. We have tried our level best to improve this manuscript by organizing and adding more references to the available relevant literature, and by presenting the material more constructively and effectively. This has certainly raised the quality of this research work and will make it a valuable contribution to the community. We hope that we have managed to make the required changes suggested by the valuable reviewers.

Please see our responses in line with the editor’s and reviewers’ concerns in green color.

Best regards,

Junaid et al.

Response to Reviewers

Reviewer #1

**Reviewer#1, Concern:** Thank you for submitting the revised version of your manuscript.
The revisions have been reviewed and are acceptable.

**Author response:** Thank you for your valuable and acknowledgeable comment. We are very grateful that all the concerns of reviewer 1 have been addressed and acknowledged.

**Author action:** All the changes have already been implemented in the submitted manuscript.

_____________________________________________________________________________________

Reviewer #2

**Reviewer#2, Concern1:** Minor Points for Addition or Clarification

Experimental Environment Details (Supplement to Concern #2):

**Author response:** Thank you for your valuable comment. As per the reviewer minor change suggestion for experimental environment details. We acknowledge the suggestion and added the more detail of experimental environment details in the updated manuscript.

**Author action:** We have updated the manuscript under section Experiments and Results. The change has been implemented in the updated manuscript.

_____________________________________________________________________________________

**Reviewer#2, Concern2:** Presentation of Algorithms and Pseudocode

**Author response:** Thank you for your valuable comment. As per the reviewer’s, the presentation of algorithms and Pseudocode modify. We acknowledge the suggestion and modify the presentation of all algorithms and pseudocodes used in the article.

**Author action:** We have updated the manuscript and modified the presentation of all algorithms and pseudocodes used in the updated manuscript.

**Reviewer#2, Concern3:** Current Status: The newly added Algorithms 1, 2, and 3 are excellent additions. However, the formatting of Algorithm 1 shows abnormal line breaks in the PDF

**Author response:** Thank you for your valuable comment. As per the reviewer’s suggestion the formatting of algorithms and Pseudocode modify. We acknowledge the suggestion and modify the formatting and abnormal breaks of all algorithms and pseudocodes used in the article.

**Author action:** We have updated the manuscript and modified the formatting of all algorithms and pseudocodes used in the updated manuscript.

Final Check for Figure/Table Reference Consistency (Concern #6)

**Reviewer#2, Concern4:** Final Check for Figure/Table Reference Consistency (Concern #6)

**Author response:** Thank you for your valuable comment. As per the reviewer’s suggestion and comment all figures and tables references consistently to be checked. We acknowledge the suggestion and modify where needed any correction against the references of any table and figure. For this specifically New block latency performance is compared in Fig 3," while the context suggests it should be "Fig 11." Is changed and corrected in the updated manuscript.

**Author action:** We have updated the manuscript and carefully modified references of tables and figures where needed in the updated manuscript.
