# Supplementary material for: Enhancing traceability and trust in the sugarcane supply chain using a blockchain-based framework with smart contracts and tokenization
Source: PLoS One. 2026 Aug 4;21(8):e0352021. doi: 10.1371/journal.pone.0352021 (PMC13436780; doi:10.1371/journal.pone.0352021)
Supplement: Peer review history

# Peer review: Decision Letter 0

**DOI:** 10.1371/journal.pone.0352021.r001

**Authors:** Marcello Stanco

9 Dec 2025

PONE-D-25-25482

Enhancing Traceability and Trust in the Sugarcane Supply Chain Using a Blockchain-Based Framework with Smart Contracts and Tokenization

PLOS One

Dear Dr. Shahaab,

Thank you for submitting your manuscript to PLOS ONE. After careful consideration, we feel that it has merit but does not fully meet PLOS ONE’s publication criteria as it currently stands. Therefore, we invite you to submit a revised version of the manuscript that addresses the points raised during the review process.

==============================

More specifically:

- The abstract and introduction must be reorganized. The abstract must be more precise, and the introduction must be rewritten to avoid overlap with the literature review.

- The methodology must be enriched with more technical details suggested by the reviewers.

- A discussion section must be added to compare the results with similar studies.

- The literature review must be more systematic.

==============================

Please submit your revised manuscript by Jan 23 2026 11:59PM. If you will need more time than this to complete your revisions, please reply to this message or contact the journal office at plosone@plos.org. When you're ready to submit your revision, log on to https://www.editorialmanager.com/pone/ and select the 'Submissions Needing Revision' folder to locate your manuscript file.

Please include the following items when submitting your revised manuscript:

- A rebuttal letter that responds to each point raised by the academic editor and reviewer(s). You should upload this letter as a separate file labeled 'Response to Reviewers'.
- A marked-up copy of your manuscript that highlights changes made to the original version. You should upload this as a separate file labeled 'Revised Manuscript with Track Changes'.
- An unmarked version of your revised paper without tracked changes. You should upload this as a separate file labeled 'Manuscript'.

If you would like to make changes to your financial disclosure, please include your updated statement in your cover letter. Guidelines for resubmitting your figure files are available below the reviewer comments at the end of this letter.

If applicable, we recommend that you deposit your laboratory protocols in protocols.io to enhance the reproducibility of your results. Protocols.io assigns your protocol its own identifier (DOI) so that it can be cited independently in the future. For instructions see: https://journals.plos.org/plosone/s/submission-guidelines#loc-laboratory-protocols. Additionally, PLOS ONE offers an option for publishing peer-reviewed Lab Protocol articles, which describe protocols hosted on protocols.io. Read more information on sharing protocols at https://plos.org/protocols?utm_medium=editorial-email&utm_source=authorletters&utm_campaign=protocols.

We look forward to receiving your revised manuscript.

Kind regards,

Marcello Stanco, Ph.D.

Academic Editor

PLOS One

Journal Requirements:

When submitting your revision, we need you to address these additional requirements.

1. Please ensure that your manuscript meets PLOS ONE's style requirements, including those for file naming. The PLOS ONE style templates can be found at

https://journals.plos.org/plosone/s/file?id=wjVg/PLOSOne_formatting_sample_main_body.pdf and

https://journals.plos.org/plosone/s/file?id=ba62/PLOSOne_formatting_sample_title_authors_affiliations.pdf

2. Please note that PLOS ONE has specific guidelines on code sharing for submissions in which author-generated code underpins the findings in the manuscript. In these cases, we expect all author-generated code to be made available without restrictions upon publication of the work. Please review our guidelines at https://journals.plos.org/plosone/s/materials-and-software-sharing#loc-sharing-code and ensure that your code is shared in a way that follows best practice and facilitates reproducibility and reuse.

3. When completing the data availability statement of the submission form, you indicated that you will make your data available on acceptance. We strongly recommend all authors decide on a data sharing plan before acceptance, as the process can be lengthy and hold up publication timelines. Please note that, though access restrictions are acceptable now, your entire data will need to be made freely accessible if your manuscript is accepted for publication. This policy applies to all data except where public deposition would breach compliance with the protocol approved by your research ethics board. If you are unable to adhere to our open data policy, please kindly revise your statement to explain your reasoning and we will seek the editor's input on an exemption. Please be assured that, once you have provided your new statement, the assessment of your exemption will not hold up the peer review process.

4. Please upload a copy of Supporting Information Figure/Table/etc. “S1 DataSet Dataset csv file).” which you refer to in your text on page 18.

5. If the reviewer comments include a recommendation to cite specific previously published works, please review and evaluate these publications to determine whether they are relevant and should be cited. There is no requirement to cite these works unless the editor has indicated otherwise.

[Note: HTML markup is below. Please do not edit.]

Reviewers' comments:

Reviewer's Responses to Questions

**Comments to the Author**

1. Is the manuscript technically sound, and do the data support the conclusions?

The manuscript must describe a technically sound piece of scientific research with data that supports the conclusions. Experiments must have been conducted rigorously, with appropriate controls, replication, and sample sizes. The conclusions must be drawn appropriately based on the data presented.

Reviewer #1: Yes

Reviewer #2: Yes

**********

2. Has the statistical analysis been performed appropriately and rigorously?

Reviewer #1: N/A

Reviewer #2: Yes

**********

3. Have the authors made all data underlying the findings in their manuscript fully available?

The PLOS Data policy requires authors to make all data underlying the findings described in their manuscript fully available without restriction, with rare exception (please refer to the Data Availability Statement in the manuscript PDF file). The data should be provided as part of the manuscript or its supporting information, or deposited to a public repository. For example, in addition to summary statistics, the data points behind means, medians and variance measures should be available. If there are restrictions on publicly sharing data—e.g. participant privacy or use of data from a third party—those must be specified.

Reviewer #1: No

Reviewer #2: Yes

**********

4. Is the manuscript presented in an intelligible fashion and written in standard English?

PLOS ONE does not copyedit accepted manuscripts, so the language in submitted articles must be clear, correct, and unambiguous. Any typographical or grammatical errors should be corrected at revision, so please note any specific errors here.

Reviewer #1: Yes

Reviewer #2: Yes

**********

5. Review Comments to the Author

Please use the space provided to explain your answers to the questions above. You may also include additional comments for the author, including concerns about dual publication, research ethics, or publication ethics. (Please upload your review as an attachment if it exceeds 20,000 characters)

Reviewer #1: Dear authors, Thank you. Please read bellow comments.

#The abstract is too long, especially its introductory part. This section should be shortened and focused on presenting the paper’s contributions, novelty, and implications.

#The writing style of the introduction resembles a literature review and needs to be rewritten. Many of these parts can be included under the Literature Review section and combined with the Related Work.

##Add a Methodology section to the paper. The research methodology requires explanation and clarification.

#Add a Discussion section and compare your findings with those of similar studies.

#Rewrite the Conclusion section. It should include research limitations and future recommendations, organized under a separate subsection.

#Provide a detailed statement of the paper’s contributions and implications.

You may prepare a table for the Literature Review section and summarize some relevant studies along with brief critical remarks. Such as : https://doi.org/10.1007/s10660-023-09696-3

Reviewer #2: I. Major Advantages

Problem Awareness: Clearly identifies issues in the sugarcane supply chain, such as lack of transparency, price volatility, and exploitation by intermediaries, demonstrating significant practical relevance.

Complete Framework Design: Proposes a comprehensive solution covering technical architecture, economic models, token issuance, and transaction processes, presenting a well-rounded system.

Reasonable Technology Integration: Effectively combines technologies like blockchain, smart contracts, IPFS, and DApps, exhibiting technological foresight.

Sufficient Experimental Validation: Supports claims with robust experimental results comparing performance in transaction latency, throughput, and fees, providing solid data backing.

Clear Structure: Well-organized sections with logical flow, enhanced by visuals, making the paper easy to understand.

II. Issues and Recommendations

1.Methodology Section Needs Further Detailing:

Specific code or pseudocode for smart contracts is not presented. It is recommended to supplement with the implementation logic of key functions or flowcharts.

The IPFS storage mechanism and data encryption methods are described too broadly. It is recommended to specify the encryption algorithms, key management strategies, and data access control mechanisms.

2.Experimental Comparison Could Be More Comprehensive:

Currently, comparisons are only made with a few blockchain systems (e.g., Bitcoin, Ethereum). It is recommended to include comparative analysis with recent agricultural blockchain projects (e.g., AgriDigital, IBM Food Trust).

Details such as the number of nodes and network topology are not mentioned in the experimental environment. It is recommended to supplement these for enhanced reproducibility.

3.Economic Model and Policy Feasibility:

The pricing mechanism for Sugar Coin (1 SC = 100 USD) lacks strategies for dealing with market fluctuations. It is recommended to discuss price stabilization mechanisms (e.g., pegged reserves, algorithmic stablecoins).

The text mentions "government-supervised transactions" but does not specify the regulatory mechanisms and compliance designs. It is recommended to supplement this.

4.User Adoption and Implementation Barriers:

The acceptance by non-technical users such as farmers and SMEs, training costs, and equipment requirements are not discussed sufficiently.

International cross-border transactions are mentioned only as future work. However, issues like tariffs, exchange rates, and compliance need to be considered in advance for practical application.

5.Literature Review Could Be More Systematic:

The review of related work on blockchain applications in agricultural supply chains could be further structured by classifying and summarizing based on technology type (traceability, payments, contracts, etc.) or crop type.

6.Formatting and Expression Details:

Some figure and table numbers and their references are inconsistent (e.g., mentions of Fig. 3, Fig. 4, etc., in the text but not fully displayed in the submitted content).

Reference formatting needs uniformity (e.g., author name abbreviations, journal name abbreviations).

**********

6. PLOS authors have the option to publish the peer review history of their article (what does this mean?). If published, this will include your full peer review and any attached files.

If you choose “no”, your identity will remain anonymous but your review may still be made public.

**Do you want your identity to be public for this peer review?**  For information about this choice, including consent withdrawal, please see our Privacy Policy.

Reviewer #1: No

Reviewer #2: No

**********

[NOTE: If reviewer comments were submitted as an attachment file, they will be attached to this email and accessible via the submission site. Please log into your account, locate the manuscript record, and check for the action link "View Attachments". If this link does not appear, there are no attachment files.]

To ensure your figures meet our technical requirements, please review our figure guidelines: https://journals.plos.org/plosone/s/figures

You may also use PLOS’s free figure tool, NAAS, to help you prepare publication quality figures: https://journals.plos.org/plosone/s/figures#loc-tools-for-figure-preparation.

NAAS will assess whether your figures meet our technical requirements by comparing each figure against our figure specifications.

# Author comment: Author response to Decision Letter 1

**DOI:** 10.1371/journal.pone.0352021.r002

27 Jan 2026

Dear Reviewers,

First, we are extremely grateful to the editor and all the reviewers who conducted a rigorous and constructive review of our article . Certainly, all the review points were very well directed, and we managed to improve our manuscript a great deal and had to work a lot to address all the changes suggested by the reviewers.

We have incorporated all the suggested reviews and comments provided by the reviewers and made a careful revision of the manuscript. We have tried our level best to improve this manuscript by organizing and adding more references to the available relevant literature, and by presenting the material more constructively and effectively. This has certainly raised the quality of this research work and will make it a valuable contribution to the community. We hope that we have managed to make the required changes suggested by the valuable reviewers.

Please see our responses in line with the editor’s and reviewers’ concerns in green color.

Best regards,

Farooq et al.

Attachment Submitted filename: Response to Reviewers.docx

# Peer review: Decision Letter 1

**DOI:** 10.1371/journal.pone.0352021.r003

**Authors:** Marcello Stanco

10 Feb 2026

PONE-D-25-25482R1

Enhancing Traceability and Trust in the Sugarcane Supply Chain Using a Blockchain-Based Framework with Smart Contracts and Tokenization

PLOS One

Dear Dr. Shahaab,

Thank you for submitting your manuscript to PLOS ONE. After careful consideration, we feel that it has merit but does not fully meet PLOS ONE’s publication criteria as it currently stands. Therefore, we invite you to submit a revised version of the manuscript that addresses the points raised during the review process.

Please submit your revised manuscript by Mar 27 2026 11:59PM. If you will need more time than this to complete your revisions, please reply to this message or contact the journal office at plosone@plos.org. When you're ready to submit your revision, log on to https://www.editorialmanager.com/pone/ and select the 'Submissions Needing Revision' folder to locate your manuscript file.

Please include the following items when submitting your revised manuscript:

- A letter that responds to each point raised by the academic editor and reviewer(s). You should upload this letter as a separate file labeled 'Response to Reviewers'.
- A marked-up copy of your manuscript that highlights changes made to the original version. You should upload this as a separate file labeled 'Revised Manuscript with Track Changes'.
- An unmarked version of your revised paper without tracked changes. You should upload this as a separate file labeled 'Manuscript'.

-->If you would like to make changes to your financial disclosure, please include your updated statement in your cover letter. Guidelines for resubmitting your figure files are available below the reviewer comments at the end of this letter.

If applicable, we recommend that you deposit your laboratory protocols in protocols.io to enhance the reproducibility of your results. Protocols.io assigns your protocol its own identifier (DOI) so that it can be cited independently in the future. For instructions see: https://journals.plos.org/plosone/s/submission-guidelines#loc-laboratory-protocols. Additionally, PLOS ONE offers an option for publishing peer-reviewed Lab Protocol articles, which describe protocols hosted on protocols.io. Read more information on sharing protocols at https://plos.org/protocols?utm_medium=editorial-email&utm_source=authorletters&utm_campaign=protocols.

We look forward to receiving your revised manuscript.

Kind regards,

Marcello Stanco, Ph.D.

Academic Editor

PLOS One

Journal Requirements:

1. If the reviewer comments include a recommendation to cite specific previously published works, please review and evaluate these publications to determine whether they are relevant and should be cited. There is no requirement to cite these works unless the editor has indicated otherwise.

2. Please review your reference list to ensure that it is complete and correct. If you have cited papers that have been retracted, please include the rationale for doing so in the manuscript text, or remove these references and replace them with relevant current references. Any changes to the reference list should be mentioned in the rebuttal letter that accompanies your revised manuscript. If you need to cite a retracted article, indicate the article’s retracted status in the References list and also include a citation and full reference for the retraction notice.

Additional Editor Comments:

Reviewer 2 recommends additional changes that will further improve the quality of the manuscript.

Therefore, I ask you to carefully implement the suggested changes.

[Note: HTML markup is below. Please do not edit.]

Reviewer's Responses to Questions

**Comments to the Author**

1. If the authors have adequately addressed your comments raised in a previous round of review and you feel that this manuscript is now acceptable for publication, you may indicate that here to bypass the “Comments to the Author” section, enter your conflict of interest statement in the “Confidential to Editor” section, and submit your "Accept" recommendation.

Reviewer #1: All comments have been addressed

Reviewer #2: All comments have been addressed

**********

2. Is the manuscript technically sound, and do the data support the conclusions?

The manuscript must describe a technically sound piece of scientific research with data that supports the conclusions. Experiments must have been conducted rigorously, with appropriate controls, replication, and sample sizes. The conclusions must be drawn appropriately based on the data presented.

Reviewer #1: (No Response)

Reviewer #2: Yes

**********

3. Has the statistical analysis been performed appropriately and rigorously?

Reviewer #1: Yes

Reviewer #2: Yes

**********

4. Have the authors made all data underlying the findings in their manuscript fully available?

The PLOS Data policy requires authors to make all data underlying the findings described in their manuscript fully available without restriction, with rare exception (please refer to the Data Availability Statement in the manuscript PDF file). The data should be provided as part of the manuscript or its supporting information, or deposited to a public repository. For example, in addition to summary statistics, the data points behind means, medians and variance measures should be available. If there are restrictions on publicly sharing data—e.g. participant privacy or use of data from a third party—those must be specified.

Reviewer #1: Yes

Reviewer #2: Yes

**********

5. Is the manuscript presented in an intelligible fashion and written in standard English?

PLOS ONE does not copyedit accepted manuscripts, so the language in submitted articles must be clear, correct, and unambiguous. Any typographical or grammatical errors should be corrected at revision, so please note any specific errors here.

Reviewer #1: Yes

Reviewer #2: Yes

**********

6. Review Comments to the Author

Please use the space provided to explain your answers to the questions above. You may also include additional comments for the author, including concerns about dual publication, research ethics, or publication ethics. (Please upload your review as an attachment if it exceeds 20,000 characters)

Reviewer #1: Dear Authors,

Thank you for submitting the revised version of your manuscript.

The revisions have been reviewed and are acceptable.

Best regards.

Reviewer #2: Decision: Minor Revision

Reason:

The authors have adequately addressed all core issues from the previous review. The manuscript's current scientific rigor, completeness, and clarity meet the publication standards. The following suggestions are only minor refinements to perfect the manuscript and do not affect the affirmation of its core value.

Detailed Comments for the Editor and Authors

Firstly, sincere congratulations to the author team on completing a high-quality revision. Your serious consideration and thorough supplementation in response to all review comments have greatly enhanced the value of this paper. The following are final suggestions for perfection:

1. Minor Points for Addition or Clarification

Experimental Environment Details (Supplement to Concern #2):

Suggestion: Please add more specific information about the test network setup at the beginning of the "Experiments and Results" section. For example: "This study deployed [please specify the number, e.g., 5] validator nodes on the Binance Smart Chain testnet, interconnected in a [please briefly describe topology, e.g., full mesh or star] topology to simulate a consortium chain environment." This would significantly enhance the reproducibility of the experiments.

Presentation of Algorithms and Pseudocode (Optimization for Concern #1):

Current Status: The newly added Algorithms 1, 2, and 3 are excellent additions. However, the formatting of Algorithm 1 shows abnormal line breaks in the PDF (e.g., "andtradedetailstoup- oaderdetails"), affecting readability.

Suggestion: Before final submission, please carefully check and correct the layout of all pseudocode to ensure it is clear, uniformly formatted, and free of word-splitting errors.

Final Check for Figure/Table Reference Consistency (Concern #6):

Current Status: Generally improved. However, for instance, the text states "New block latency performance is compared in Fig 3," while the context suggests it should be "Fig 11." Such minor inconsistencies might still remain.

Suggestion: Before submitting the final version, conduct a thorough cross-verification from text to all figures and tables to ensure every citation of "Fig. X" and "Table X" is accurate.

2. Evaluation of the Authors' Response

Concern #1 & #3 (Methodology & Economic Model): The response far exceeded expectations. Not only was pseudocode provided, but encryption strategies and price stabilization mechanisms were also detailed—one of the greatest highlights of this revision.

Concern #2 (Experimental Comparison): The newly added Tables 8 and 9 are excellent, clearly positioning this study within the field of agricultural blockchain.

Concern #4 & #5 (User Adoption & Literature): The supplemented discussion and tables have enriched the practical and academic value of the paper.

Concern #6 & #7 (Formatting & Transparency): These have been properly addressed.

3. Final Decision and Closing Remarks

This manuscript, through this round of careful revision, has transformed from a paper with potential into a solid and comprehensive contribution to the field. It not only proposes a detailed technical solution but also, commendably, delves into critical practical issues such as economic modeling, regulatory compliance, and user adoption for real-world implementation.

It is recommended that the editor accept this paper after the authors complete the aforementioned minor corrections. These corrections do not affect the main content or scientific conclusions of the manuscript, only concerning clarity of expression and detail completeness.

To the Authors: Your work is impressive. Please perform a final round of proofreading and minor adjustments based on the above suggestions. We look forward to seeing the formal publication of this excellent paper in PLOS ONE.

**********

7. PLOS authors have the option to publish the peer review history of their article (what does this mean?). If published, this will include your full peer review and any attached files.

If you choose “no”, your identity will remain anonymous but your review may still be made public.

**Do you want your identity to be public for this peer review?**  For information about this choice, including consent withdrawal, please see our Privacy Policy.

Reviewer #1: No

Reviewer #2: No

**********

[NOTE: If reviewer comments were submitted as an attachment file, they will be attached to this email and accessible via the submission site. Please log into your account, locate the manuscript record, and check for the action link "View Attachments". If this link does not appear, there are no attachment files.]

To ensure your figures meet our technical requirements, please review our figure guidelines: https://journals.plos.org/plosone/s/figures

You may also use PLOS’s free figure tool, NAAS, to help you prepare publication quality figures: https://journals.plos.org/plosone/s/figures#loc-tools-for-figure-preparation.

NAAS will assess whether your figures meet our technical requirements by comparing each figure against our figure specifications.

# Author comment: Author response to Decision Letter 2

**DOI:** 10.1371/journal.pone.0352021.r004

1 May 2026

We have acted on the reviewer 2's feedback and made amends as requested.

Attachment Submitted filename: Response to Reviewers R2.docx

# Editor report: Decision Letter 2

**DOI:** 10.1371/journal.pone.0352021.r005

**Authors:** Marcello Stanco

4 Jun 2026

Enhancing Traceability and Trust in the Sugarcane Supply Chain Using a Blockchain-Based Framework with Smart Contracts and Tokenization

PONE-D-25-25482R2

Dear Dr. Shahaab,

We’re pleased to inform you that your manuscript has been judged scientifically suitable for publication and will be formally accepted for publication once it meets all outstanding technical requirements.

Within one week, you’ll receive an e-mail detailing the required amendments. When these have been addressed, you’ll receive a formal acceptance letter and your manuscript will be scheduled for publication.

An invoice will be generated when your article is formally accepted. Please note, if your institution has a publishing partnership with PLOS and your article meets the relevant criteria, all or part of your publication costs will be covered. Please make sure your user information is up-to-date by logging into Editorial Manager at Editorial Manager® and clicking the ‘Update My Information' link at the top of the page. For questions related to billing, please contact billing support.

If your institution or institutions have a press office, please notify them about your upcoming paper to help maximize its impact. If they’ll be preparing press materials, please inform our press team as soon as possible -- no later than 48 hours after receiving the formal acceptance. Your manuscript will remain under strict press embargo until 2 pm Eastern Time on the date of publication. For more information, please contact onepress@plos.org.

Kind regards,

Marcello Stanco, Ph.D.

Academic Editor

PLOS One

Additional Editor Comments (optional):

Reviewers' comments:

# Editor report: Acceptance letter

**DOI:** 10.1371/journal.pone.0352021.r006

**Authors:** Marcello Stanco

PONE-D-25-25482R2

PLOS One

Dear Dr. Shahaab,

I'm pleased to inform you that your manuscript has been deemed suitable for publication in PLOS One. Congratulations! Your manuscript is now being handed over to our production team.

At this stage, our production department will prepare your paper for publication. This includes ensuring the following:

* All references, tables, and figures are properly cited

* All relevant supporting information is included in the manuscript submission,

* There are no issues that prevent the paper from being properly typeset

You will receive further instructions from the production team, including instructions on how to review your proof when it is ready. Please keep in mind that we are working through a large volume of accepted articles, so please give us a few days to review your paper and let you know the next and final steps.

Lastly, if your institution or institutions have a press office, please let them know about your upcoming paper now to help maximize its impact. If they'll be preparing press materials, please inform our press team within the next 48 hours. Your manuscript will remain under strict press embargo until 2 pm Eastern Time on the date of publication. For more information, please contact onepress@plos.org.

You will receive an invoice from PLOS for your publication fee after your manuscript has reached the completed accept phase. If you receive an email requesting payment before acceptance or for any other service, this may be a phishing scheme. Learn how to identify phishing emails and protect your accounts at https://explore.plos.org/phishing.

If we can help with anything else, please email us at customercare@plos.org.

Thank you for submitting your work to PLOS ONE and supporting open access.

Kind regards,

PLOS ONE Editorial Office Staff

on behalf of

Dr. Marcello Stanco

Academic Editor

PLOS One
